# Exercise intervention for patients with chronic low back pain: a systematic review and network meta-analysis

**DOI:** 10.3389/fpubh.2023.1155225

**Published:** 2023-11-17

**Authors:** Ying Li, Lei Yan, Lingyu Hou, Xiaoya Zhang, Hanping Zhao, Chengkun Yan, Xianhuang Li, Yuanhe Li, Xiaoan Chen, Xiaorong Ding

**Affiliations:** ^1^College of Sports Science, Jishou University, Jishou, Hunan, China; ^2^Department of Orthopaedic Surgery, Shanxi Medical University Second Affiliated Hospital, Taiyuan, China; ^3^Second Clinical Medical College, Shanxi Medical University, Taiyuan, China; ^4^Department of Nursing, Peking University Shenzhen Hospital, Shenzhen, China; ^5^School of Nursing, Nanjing University of Chinese Medicine, Nanjing, Jiangsu, China; ^6^College of Nursing, Weifang University of Science and Technology, Weifang, Shandong, China; ^7^School of Nursing, Nanchang University, Nanchang, Jiangxi, China; ^8^Digestive Endoscopy Center, The First Affiliated Hospital of Nanchang University, Nanchang, Jiangxi, China

**Keywords:** exercise therapy, chronic low back pain, network meta-analysis, CLBP, aging and public health

## Abstract

**Purpose:**

Chronic low back pain (CLBP) is an aging and public health issue that is a leading cause of disability worldwide and has a significant economic impact on a global scale. Treatments for CLBP are varied, and there is currently no study with high-quality evidence to show which treatment works best. Exercise therapy has the characteristics of minor harm, low cost, and convenient implementation. It has become a mainstream treatment method in clinics for chronic low back pain. However, there is insufficient evidence on which specific exercise regimen is more effective for chronic non-specific low back pain. This network meta-analysis aimed to evaluate the effects of different exercise therapies on chronic low back pain and provide a reference for exercise regimens in CLBP patients.

**Methods:**

We searched PubMed, EMBASE, Cochrane Library, and Web of Science from inception to 10 May 2022. Inclusion and exclusion criteria were used for selection. We collected information from studies to compare the effects of 20 exercise interventions on patients with chronic low back pain.

**Results:**

This study included 75 randomized controlled trials (RCTs) with 5,254 participants. Network meta-analysis results showed that tai chi [standardized mean difference (SMD), −2.11; 95% CI, −3.62 to −0.61], yoga (SMD, −1.76; 95% CI −2.72 to −0.81), Pilates exercise (SMD, −1.52; 95% CI, −2.68, to −0.36), and sling exercise (SMD, −1.19; 95% CI, −2.07 to −0.30) showed a better pain improvement than conventional rehabilitation. Tai chi (SMD, −2.42; 95% CI, −3.81 to −1.03) and yoga (SMD, −2.07; 95% CI, −2.80 to −1.34) showed a better pain improvement than no intervention provided. Yoga (SMD, −1.72; 95% CI, −2.91 to −0.53) and core or stabilization exercises (SMD, −1.04; 95% CI, −1.80 to −0.28) showed a better physical function improvement than conventional rehabilitation. Yoga (SMD, −1.81; 95% CI, −2.78 to −0.83) and core or stabilization exercises (SMD, −1.13; 95% CI, −1.66 to −0.59) showed a better physical function improvement than no intervention provided.

**Conclusion:**

Compared with conventional rehabilitation and no intervention provided, tai chi, toga, Pilates exercise, sling exercise, motor control exercise, and core or stabilization exercises significantly improved CLBP in patients. Compared with conventional rehabilitation and no intervention provided, yoga and core or stabilization exercises were statistically significant in improving physical function in patients with CLBP. Due to the limitations of the quality and quantity of the included studies, it is difficult to make a definitive recommendation before more large-scale and high-quality RCTs are conducted.

## Introduction

Low back pain is a more severe low back pain syndrome that can be classified by duration as acute (pain lasting for less than 6 weeks), subchronic (6–12 weeks), or chronic (more than 12 weeks) (1, 2). Only 39–76% of patients fully recover after an acute pain episode, suggesting that a significant proportion suffer from chronic low back pain (CLBP) (3). CLBP is a common and effective public health problem worldwide and is the second most common reason for medical visits in people aged 65 years or older (4, 5). Studies have found that the incidence and prevalence of CLBP increase with age (6–8). CLBP imposes an enormous economic and social burden, which will become even more onerous in the coming decades as the number of patients with CLBP is expected to increase significantly (9). In addition, a study of nearly 200,000 people in 43 countries found that those with CLBP were twice as likely to suffer from depression, anxiety, psychosis, or sleep deprivation (10, 11).

CLBP can lead to disability, high treatment costs, absenteeism, and sick leave (10). Exercise therapy is based on kinematics, biomechanics, physiology, and pathology to improve body function, regulate physiological state, improve mental quality, and eliminate mental disorders. Exercise therapy is characterized by low harm, low cost, and easy implementation and has become the first choice in the clinical treatment of CLBP (12). There are many kinds of exercise therapy (12), and it is unclear which exercise therapy is the best. Direct comparative evidence of exercise therapy suggests that core stability training is more effective than aerobic and stretching exercises in treating CLBP (13).

A review by Hayden reported that exercise therapy might be more effective than education and non-exercise physiotherapy alone in improving pain and function (14). However, Pilates remains controversial for CLBP pain, as reported in paired meta-analyses (15). In a previous net meta-analysis, studies found that exercise and heat were the best modalities for relieving CLBP pain (16). However, we found that the current study needs a detailed breakdown of exercise modalities (16) as it does not describe the effects of all current exercise modalities, such as tai chi and water sports, on the effects of CLBP (17, 18). We wanted to better explore the effects of other exercises on CLBP patients. We performed a complex variety of exercise therapies, included more exercise modalities in our network meta-analysis, and analyzed RCTs on the effects of different exercise therapies on patients with CLBP to evaluate their therapeutic effects comprehensively and suggest the best exercise therapy for selecting exercise programs.

## Materials and methods

This network meta-analysis was designed according to the guidelines for Preferred Reporting Items of Systems Review and Network Meta-Analysis (PRISMA-NMA) (19), registered in the PROSPERO database (CRD42023388526).

### Search strategy

PubMed, Web of Science, Embase, and Cochrane Library were searched to identify studies published as of 10 May 2022 associated with RCT of exercise therapy for CLBP. The search takes a combination of subject words and free words. The search strategy is shown in Supplementary Appendix 1.

### Study selection

Two independent reviewers (Chengkun Yan and Xian Huang Li) screened the titles and abstracts of publications retrieved by the search strategy to identify those eligible for inclusion. The full text of potentially eligible studies was evaluated according to the inclusion and exclusion criteria. The disagreements between reviewers were resolved through discussion. The NoteExpress software is used to manage this phase.

### Inclusion criteria

Inclusion and exclusion criteria are based on PICOS standards, see Table 1 for specific inclusion and exclusion criteria.

**Table 1:** Inclusion and exclusion criteria.

Category	Inclusion criteria	Exclusion criteria
Population	Patients diagnosed with chronic low back pain (20, 21)	Patients with severe high blood pressure, heart disease, or other serious systemic diseases
Interventions	Core or stabilization exercises (CSE), yoga, McKenzie exercise (MKE), aerobic exercise (AE), water-based physical activity (WPA), physical therapies (PT), manual treatment (MAT), sling exercise (SE), tai chi (TC), Pilates exercise (PE), other exercise (OE), motor control exercise (MCE), muscle training (MUT), multimodal exercise (MUE), conventional rehabilitation (COR), no intervention provided (NIP), home exercise (HE), stretching exercise (STE), virtual reality exercise (VR), and education (ED).	
Comparisons	No intervention provided (NIP) and conventional rehabilitation (COR).	
Outcomes	The primary analysis for this study was to assess the intensity of the pain [Visual Analog Scale (VAS) or Numerical Rating Scale (NRS)]. The second analysis was the Roland Morris Disability Questionnaire (RMDQ) or the Oswestry Disability Index (ODI) for treatment response to back-related functional limitations.	
Study	Randomized controlled trial; published in English or Chinese	

Each intervention is defined in Supplementary Appendix 2. Each result outcome measure is defined in Supplementary Appendix 3.

### Data extraction

Data extraction pairs of reviewers independently extracted the following data: first author, year of publication, country, sample size, CLBP time, age, weight, height, intervention, and intervention time. Data were expressed as mean ± standard deviation (SD). If outcome measures report multiple time points, we extract the data for the latest time point.

### Risk of bias assessment

The risk of bias was assessed independently by two reviewers and adjudicated by a third reviewer using the Cochrane Collaboration’s tools (22), which include sequence generation, assignment hiding, blinding, incomplete results data, non-selective results reporting, and other sources of bias. Each criterion was judged to have a low, unclear, or high risk of bias.

### Data analysis

We used the “netmeta” package of R-4.2.1 software to conduct a network meta-analysis. The STATA 15.1 “networkplot” function is used to draw and generate network diagrams to describe and present different forms of exercise. We used nodes to represent various interventions and edges to represent head-to-head comparisons between interventions. The node split method was used to assess inconsistency between direct and indirect comparisons (23). The pooled estimates and 95% confidence intervals (95% CI) were calculated using random effects network element analysis. When we are interested in outcomes that use the same unit of measurement in the study, consider mean difference (MD) as a therapeutic effect to analyze the results or evaluate standardized mean difference (SMD). A pairwise random-effects meta-analysis was performed to compare various exercise treatments. Heterogeneity was assessed for all pairwise comparisons using the *I*^2^ statistic and publication bias using the value of p of Egger’s test. Funnel plots were conducted to determine publication bias and minor study effects measured by results reported in more than 10 studies.

## Results

### Literature selection

**Figure 1:**
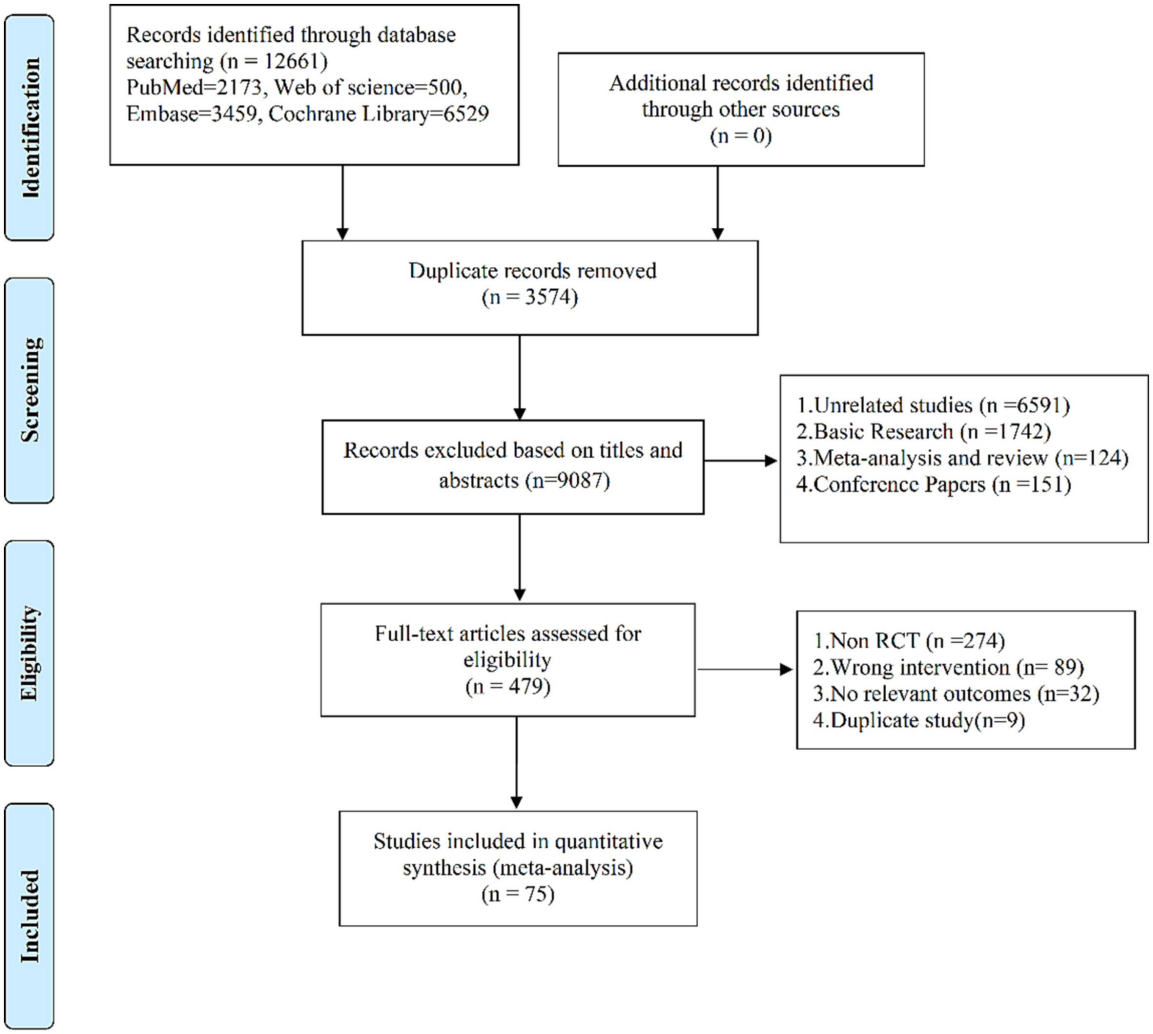
Flow of trials throughout the review.

After deleting duplicates, 9,087 records were retrieved, and 8,608 studies were discarded. The full text of the remaining 479 records was examined, and 404 records did not meet the inclusion criteria: 274 were non-RCTs, 89 were wrong interventions, 32 were no relevant outcomes, and 9 were duplicate studies. In the end, 75 studies (24–98) were included. The research flow chart is shown in Figure 1.

### Study and participant characteristics

**Figure 2:**
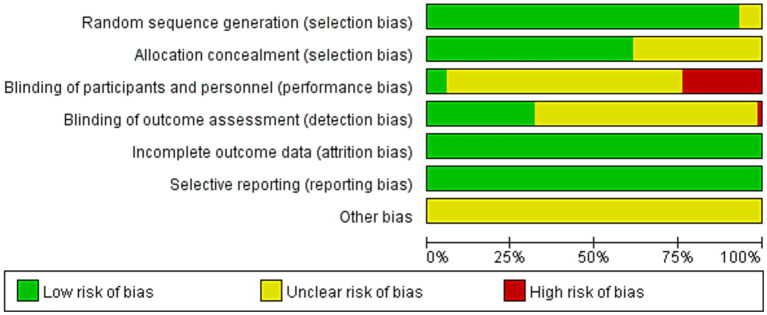
Percentage of studies examining the efficacy of exercise training in patients with non-specific chronic low back pain with low, unclear, and high risk of bias for each feature of the Cochrane Risk of Bias Tool.

The included studies, published between 1998 and 2021, compared the effects of 20 different therapies on CLBP. The intervention lasted from 7 days to 24 weeks. A total of 5,254 patients were reported in the included studies. Of all the included studies, 46 reported VAS, 16 reported NRS, 41 reported ODI, and 13 reported RMDQ. The average age ranged from 20.1 ± 0.7 to 70.4 ± 3.2 years, the average weight ranged from 54.7 ± 7.6 kg to 81 ± 18.6 kg, and the average height ranged from 156.11 ± 9.44 cm to 177.60 ± 9.98 cm. The characteristics of the studies and the participants are shown in Table 2 and Supplementary Appendix 4. The risk of bias assessment for each individual study is presented in Supplementary Appendix 5 and summary data in Figure 2. In addition, we conducted regression analyses of age and gender, as shown in Supplementary Appendix 9.

**Table 2:** General characteristics of all included studies.

Characteristics	VAS	NRS	ODI	RMDQ
Publication characteristics				
Total number of unique studies included	46	16	41	13
Publication year				
1991–2000	3	0	1	1
2001–2010	9	6	10	4
2011–2021	34	10	30	8
Study design characteristics				
Range of study sample size				
1–50	28	6	21	8
51–100	9	4	9	2
101–150	7	3	7	3
151–200	0	2	1	0
>200	2	1	3	0
No. of intervention arms included				
2	39	9	34	9
3	7	6	6	4
4	0	1	1	0
No. of studies containing the following treatment nodes				
Core or stabilization exercises (CSE)	20	5	18	2
Yoga	4	1	4	0
McKenzie exercise (MKE)	4	1	3	0
Aerobic exercise (AE)	6	0	4	3
Water-based physical activity (WPA)	1	1	1	0
Physical therapies (PT)	8	1	5	3
Manual treatment (MAT)	4	1	3	1
Sling exercise (SE)	3	2	3	0
Tai chi (TC)	2	0	0	0
Pilates exercise (PE)	2	2	2	3
Other exercise (OE)	7	7	11	1
Motor control exercise (MCE)	2	3	3	0
Muscle training (MUT)	9	2	8	5
Multimodal exercise (MUE)	7	2	7	2
No intervention provided (NIP)	12	7	10	9
Conventional rehabilitation (COR)	5	1	4	0
Home exercise (HE)	1	1	1	1
Stretching exercise (STE)	1	0	0	0
Virtual reality (VR)	0	1	1	0
Education (ED)	1	2	2	0
Time of intervention				
Unclear	0	0	0	0
7 days	1	0	1	0
10 days	1	0	1	0
4 weeks	10	6	10	2
6 weeks	11	1	11	1
8 weeks	9	7	9	4
12 weeks	9	2	7	5
13 weeks	1	0	0	0
16 weeks	1	0	0	0
20 weeks	1	0	1	0
24 weeks	1	0	1	0
6 months	1	0	0	1
12 months	0	0	0	0
Intervention frequency				
1 times/week	0	2	3	1
2 times/week	12	2	8	5
3 times/week	18	4	14	2
4 times/week	1	0	2	1
5 times/week	4	2	4	0
7 times/week	1	0	0	0
Unclear	10	6	10	4
Countries				
Turkey	5	1	5	1
Korea	15	2	9	1
Greece	0	3	2	2
Brazil	1	1	2	1
Israel	2	0	1	2
Thailand	0	1	1	0
Australia	0	2	1	0
USA	3	1	2	0
Netherlands	0	0	0	1
Canada	2	2	4	0
Spain	2	0	2	1
Iran	5	0	2	1
Germany	1	0	0	0
Egypt	0	0	0	1
Lithuania	1	0	1	0
Pakistan	0	0	1	0
India	1	0	1	0
Poland	1	0	1	0
Kosovo	2	0	2	0
Norway	1	1	2	0
Singapore	0	1	0	0
Croatia	0	1	1	0
China	1	0	0	0
Finland	3	0	0	2
Austria	0	0	1	0
Patient characteristics				
Range of mean age (years)	20.1–70.4	26.0–68.8	26.0–68.8	23.45–57.19
Range of mean weight (kg)	54.7–82.3	55.6–80.8	55.6–80.8	63.3–80.1
Range of mean height (cm)	156.55–177	160–172.6	156.11–177.60	161.21–173

#### Outcomes

##### Pain

**Figure 3:**
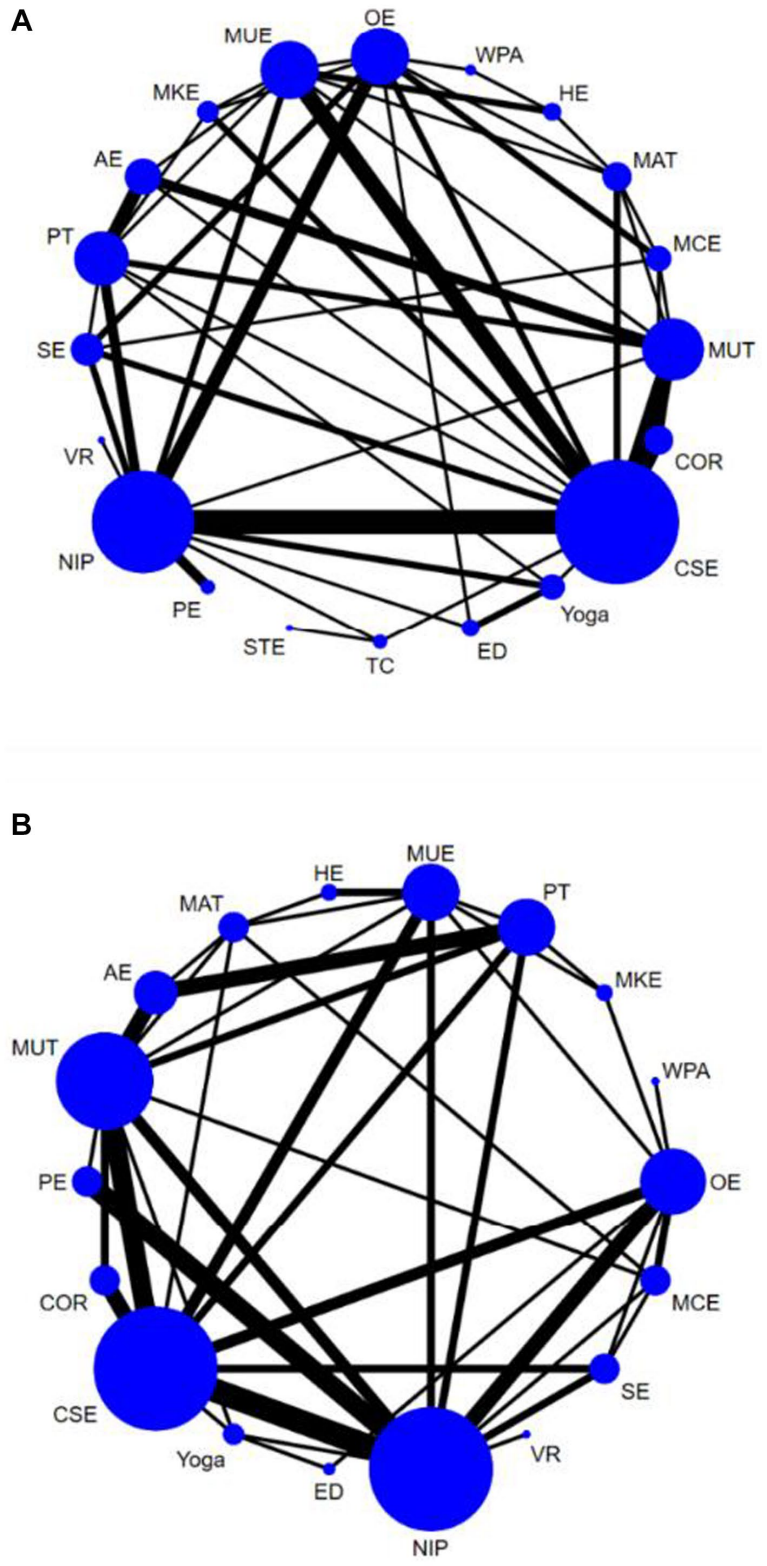
Network plots of pain and Physical function. The size of the nodes represents how many times the exercise appears in any comparison aboutthat treatment and the width of the edges represents the total sample size in the comparisons it connects. Core or stabilization exercises (CSE), Yoga, McKenzie exercise (MKE), Aerobic exercise (AE), Water-based physical activity (WPA), Physical therapies (PT), Manual treatment (MAT), Sling exercise (SE), Tai chi (TC), Pilates exercise (PE), Other exercise (OE), Motor control exercise (MCE), Muscle training (MUT), Multimodal exercise (MUE), Conventional rehabilitation (COR), No intervention provided (NIP), Home exercise (HE), Stretching exercise (STE).Virtual Reality exercise (VR), Education (ED).

In total, 62 studies (24–30, 33, 35–50, 52–56, 58, 60, 61, 63–65, 67–71, 73–79, 82–84, 86–92, 94–98) assessed pain, involving a total of 3,123 participants. We included the following 20 interventions in our network meta-analysis (Figure 2): TC, yoga, PE, SE, MCE, WPA, CSE, MUE, MKE, HE, MAT, MUT, STE, ED, OE, AE, PT, VR, COR, and NIP. TC (SMD, −2.11; 95% CI, −3.62 to −0.61), yoga (SMD, −1.76; 95% CI, −2.72 to −0.81), PE (SMD, −1.52; 95% CI, −2.68, to −0.36), SE (SMD, −1.19; 95% CI, −2.07 to −0.30), MCE (SMD, −1.02; 95% CI, −1.86 to −0.18), CSE (SMD, −0.95; 95% CI, −1.56 to −0.33), MUE (SMD, −0.94; 95% CI, −1.71 to −0.18), and MKE (SMD, −0.91; 95% CI, −1.81 to −0.01) showed a better pain improvement than COR. TC (SMD, −2.42; 95% CI, −3.81 to −1.03), yoga (SMD, −2.07; 95% CI, −2.80 to −1.34), PE (SMD, −1.83; 95% CI, −2.72 to −0.93), SE (SMD, −1.49; 95% CI, −2.20 to −0.79), MCE (SMD, −1.33; 95% CI, −2.12 to −0.54), WPA (SMD, −1.36; 95% CI, −2.59 to −0.14), CSE (SMD, −1.25; 95% CI, −1.71 to −0.79), MUE (SMD, −1.25; 95% CI, −1.84 to −0.65), MKE (SMD, −1.22; 95% CI, −1.98 to −0.45), HE (SMD, −1.20; 95% CI, −2.19 to −0.22), MAT (SMD, −1.16; 95% CI, −1.89 to −0.42), MUT (SMD, −1.08; 95% CI, −1.67 to −0.49), STE (SMD, −1.02; 95% CI, −3.07 to −1.03), ED (SMD, −0.93; 95% CI, −1.84 to −0.01), OE (SMD, −0.97; 95% CI, −1.52 to −0.43), and AE (SMD, −0.82; 95% CI, −1.54 to −0.10) showed a better pain improvement than NIP (Figure 3A). The comparison adjusted funnel plot did not provide evidence for apparent publication bias and Egger’s test (*p* = 0.601) (Supplementary Appendix 6.1). Heterogeneity, intransitivity, and inconsistency of the network meta-analysis were also evaluated (Supplementary Appendix 7). Direct pain was also evaluated (Supplementary Appendix 8.1).

### Physical function

**Figure 4:**
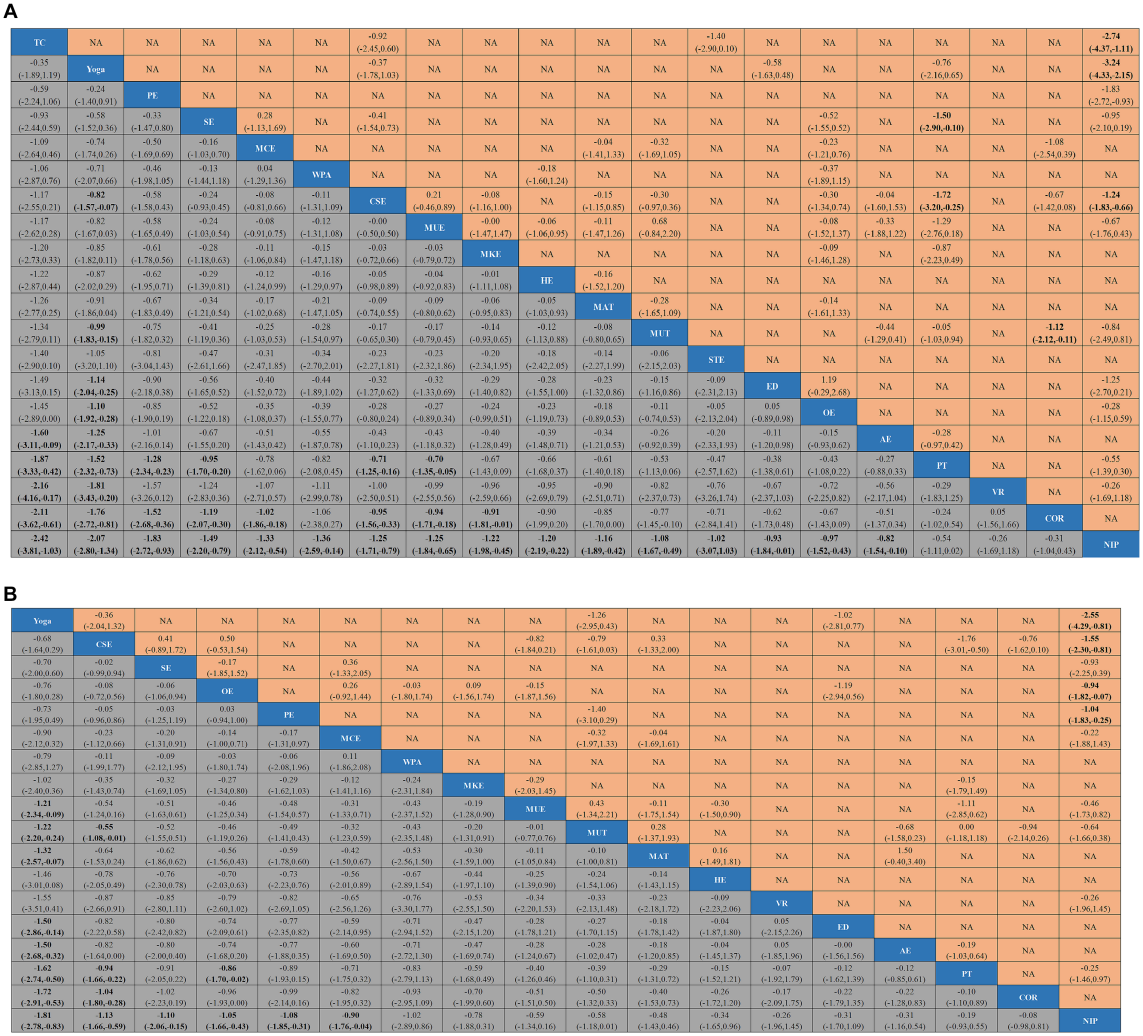
League tables of outcome analyses. Data are mean differences and 95% credibility intervals for continuous data. Core or stabilization exercises (CSE), Yoga, McKenzie exercise (MKE), Aerobic exercise (AE), Water-based physical activity (WPA), Physical therapies (PT), Manual treatment (MAT), Sling exercise (SE), Tai chi (TC), Pilates exercise (PE), Other exercise (OE), Motor control exercise (MCE), Muscle training (MUT), Multimodal exercise (MUE), Conventional rehabilitation (COR), No intervention provided (NIP), Home exercise (HE), Stretching exercise (STE).Virtual Reality exercise (VR), Education (ED).

In total, 54 studies (28, 30–35, 38, 39, 41, 44–55, 57–64, 66, 67, 72, 74, 76–82, 85–88, 90–98) assessed physical function, involving a total of 4,355 participants. We included the following 18 interventions in our network meta-analysis (Figure 2), including the NIP, VR, SE, MCE, OE, WAP, MKE, PT, MUE, HE, MAT, AE, MUT, PE, COR, CSE, yoga, and ED. Yoga (SMD, −1.72; 95% CI, −2.91 to −0.53), CSE (SMD, −1.04; 95% CI, −1.80 to −0.28) showed a better physical function improvement than COR. Yoga (SMD, −1.81; 95% CI, −2.78 to −0.83), CSE (SMD, −1.13; 95% CI, −1.66 to −0.59), SE (SMD, −1.10; 95% CI, −2.06 to −0.15), OE (SMD, −1.05; 95% CI, −1.66 to −0.43), PE (SMD; −1.08, 95% CI, −1.85 to −0.31), and MCE (SMD, −0.90; 95% CI, −1.76 to −0.04) showed a better physical function improvement than NIP (Figure 3B). The comparison-adjusted funnel plot did not provide evidence for apparent publication bias and Egger’s test (*p* = 0.616) (Supplementary Appendix 6.2). Heterogeneity, intransitivity, and inconsistency of the network meta-analysis (NMA) were evaluated (Supplementary Appendix 7). Direct comparisons of physical function were also evaluated (Supplementary Appendix 8.2 and Figure 4).

## Discussion

CLBP is a global aging and public health problem. The global 1-year prevalence of CLBP in older adults is 13–50% (99, 100). The medical burden associated with CLBP is high, not only due to direct costs (medical appointments, tests, medications, and hospitalizations) but also due to loss of work productivity (101, 102). Exercise therapy can relieve pain and improve dysfunction in CLBP. Nevertheless, there are many types of exercise therapy, and it needs to be clarified which exercise is the best training method. In this study, we assessed the relative effects of 20 different interventions on pain and physical function in patients with CLBP. Regression analyses showed no correlation between patient age and patients’ pain scores and physical functioning scores. In addition, regression analyses also showed no correlation between patients’ gender and patients’ pain scores and physical function scores. Tai chi, yoga, Pilates exercise, sling exercise, motor control exercise, core or stabilization exercises, multimodal exercise, and McKenzie exercise are more beneficial for pain relief than conventional rehabilitation, and no intervention is provided. Water-based physical activity, home exercise, manual treatment, muscle training, stretching exercise, education, other exercise, and aerobic exercise are more useful for pain relief than no intervention provided. Yoga and core or stabilization exercises showed better physical function improvement than conventional rehabilitation, and no intervention was provided. Sling exercise, Pilates exercise, and motor control exercise, other exercises, showed better physical function improvement than no intervention provided.

In our study, we found that tai chi can reduce pain in patients with chronic low back pain. The results of this study are the same as those of Lauche et al. (103). Compared with other forms of exercise, tai chi can increase structural flexibility and mobility, improve muscle strength and endurance, increase the tensile strength of ligaments and bursae, enhance cardiopulmonary function, and reduce stress, anxiety, and depression (104). Tai chi can significantly increase bone density value, improve limb motor and balance function, and effectively improve the symptoms of low back pain (103, 105). In addition, CLBP trunk proprioception is diminished, resulting in deficits in the control of ankle and hip strategies during balance control, a phenomenon that exacerbates the decreased trunk proprioception in CLBP (106). Tai chi can alter brain waves in the brain’s perception of pain areas (parietal and prefrontal lobes), and the brain processes relevant information more efficiently, improving proprioception in the brain centers (107). Some studies have reported that tai chi can reduce serum B-type linalool peptide levels, increase per-pulse output, improve blood circulation throughout the body, and improved blood circulation can transport blood calcium and other nutrients to the lumbar region, increase the metabolism of the lumbar bones, improve the absorption of calcium and other minerals by bone cells, and improve bone density in the lumbar region (108). The 2017 American Medical Association’s Authoritative Guidelines for the treatment of low back pain recommend tai chi for the treatment of chronic low back pain (109). Our study further supports this result.

Physical therapies involve whole-body movement that emphasizes the body posture of the human body standing and enhances the body control and balance ability through the brain consciousness to control smooth body movements and correct breathing (110, 111). In addition to core strengthening, physical therapies emphasize the coordination of breathing and movement posture, which can reduce joint contraction and fatigue of trunk muscles, effectively reduce pain, and improve body function, and are widely used in treating CLBP.

One study reported that in patients with low back pain, the height of the intervertebral disc and the length and load of the paravertebral ligament changed, and the adaptability of the proprioceptive receptor decreased, thus reducing the proprioceptive input and weakening the neuromuscular reflex of the paravertebral muscle, resulting in lumbar instability and decreased postural control (112). Sling exercise activates the core muscle group by suspending part of the body and placing the body in an unstable state, improving muscle imbalance, improving the control ability of the neuromuscular system, enhancing the stability of the lumbar spine, and improving physical function. In our study, core stabilization training was found to be effective in reducing pain. Changes in plasma β-endorphin levels can indicate efficacy response in chronic lower back pain (113). Cortisol is a type of glucocorticoid produced by the hypothalamic–pituitary–adrenal axis activity. Uncomfortable physical pain in CLBP can trigger anxiety in patients. Pain and anxiety lead to increased hypothalamic-pituitary-adrenal axis activation, leading to elevated cortisol levels in patients (114). In addition, pain neuronal excitability releases transmitters (115). In addition, interleukin 4 (IL-4), an anti-inflammatory cytokine produced by macrophages and monocytes, inhibits the synthesis of pro-inflammatory cytokines. It has been found (114, 116–118) that the mechanism of action of core stabilization training for CLBP is mainly through altering the neurotransmitters β-endorphin, cortisol, and IL-4 levels. Ko et al. (74) proved that both suspension and stability training could effectively relieve pain and enhance lumbar muscle strength and flexibility in CLBP patients.

In our study, we found that Yoga can reduce pain in patients with chronic low back pain. The results of this study are the same as those of Zhu et al. (119). Yoga originated in India and has a history of more than 4,000 years. While promoting spinal tissue stretching, flexibility, and balance training, yoga can also strengthen back muscles, relieve pain, and improve patients with functional impairment (56). In addition, yoga can effectively improve the lumbar pain and spinal flexibility of CLBP patients by stretching the spine vertebra and making the lumbar spine get strength training. More than half of CLBP patients in the United States choose yoga as an adjunct therapy (120). Guidelines developed by the American Pain Society suggest that for CLBP not alleviated by medication and self-management, consider recommending yoga as adjunctive therapy to help patients relieve pain (121).

Fernández-Rodríguez et al. conducted a network meta-analysis of nine exercises. They found that Pilates was the most effective intervention for reducing pain (114). Unlike our study, tai chi and yoga were more effective than Pilates exercises in reducing pain. In Gianola et al. (16) and Owen et al. (18) network meta-analysis, Tai chi was not treated as a separate intervention, and conventional rehabilitation and no intervention provided were not treated as control groups. However, we have classified, in detail, the different exercises into 20 other activities, including tai chi, virtual reality exercises, and conventional rehabilitation, for a more comprehensive NMA. Our study provides evidence that “active therapies” such as tai chi, yoga, sling exercise, and core or stabilization exercises, in which patients are guided and actively encouraged to move and exercise in a gradual manner, are most effective. In our study, we did not recommend virtual reality exercise, conventional rehabilitation, and no intervention provided for CLBP patients. They are less effective in pain in patients with CLBP.

## Strengths and limitations

Our review has several strengths. First, we used the network meta-analysis design to synthesize direct and indirect evidence from various exercise interventions that can be used to treat CLBP. Importantly, we used a nuanced approach to categorize exercise interventions. Previous reviews have often grouped different exercise interventions, potentially leading to heterogeneous comparisons and inaccurate estimates of therapeutic effectiveness in a single comparison. Interventions were divided into 20 types, and various interventions were defined. However, we also have certain limitations. First, we did not take the intervention period, intensity, and frequency into consideration. Second, the implementation quality of the blind method included in the literature is not high, and pain and functional improvement are subjective indicators, which may lead to the bias of the results due to the different focus of researchers. Third, we only included English literature, which may lead to heterogeneity. Fourth, the study did not analyze differences by initial categories that are important for both VAS and physical function. Fifth, CLBP was not considered in terms of the presence of a neuropathic or nocioplastic component. Sixth, biomarkers showing the effects of different exercise interventions were unavailable in the study. Finally, gender considerations were missing from the study reports. There are some gender differences in abdominal and lumbar muscle characteristics between female and male subjects (122), which can lead to differences in response to post-exercise emerging in gender-specific CLBP patients.

## Conclusion

This systematic review examined pain reduction and physical function improvement in patients with CLBP treated with exercise. Compared to COR and NIP, exercise is effective in relieving pain and improving physical function. In conclusion, understanding the benefits of exercise versus non-exercise therapy is essential to better serve patients with CLBP.

## Author contributions

YuL and LH designed the study. CY and XL acquired, analyzed, and interpreted the data. XD, YiL, YH, and YuL revised the manuscript. XZ and XC contributed to the revision of the article. All authors contributed to the article and approved the submitted version.
