# Supplementary material for: Exercise intervention for patients with chronic low back pain: a systematic review and network meta-analysis
Source: Front Public Health. 2023 Nov 17;11:1155225. doi: 10.3389/fpubh.2023.1155225 (PMC10687566; doi:10.3389/fpubh.2023.1155225)
Supplement: Supplementary file 1 [file Data_Sheet_1.zip › Supplementary Appendix 1.DOCX]

**Pubmed:**

| Number | Search terms | Results |
| --- | --- | --- |
| **#1** | ((((((((((((((((((((((((((((Back Pain, Low[Mesh Terms]) OR (Back Pains, Low[Title/Abstract])) OR (Low Back Pain[Title/Abstract])) OR (Pain, Low Back[Title/Abstract])) OR (Pains, Low Back[Title/Abstract])) OR (Lumbago [Title/Abstract])) OR (Lower Back Pain[Title/Abstract])) OR (Back Pain, Lower[Title/Abstract])) OR (Back Pains, Lower[Title/Abstract])) OR (Lower Back Pains[Title/Abstract])) OR (Pain, Lower Back[Title/Abstract])) OR (Pains, Lower Back[Title/Abstract])) OR (Low Back Ache[Title/Abstract])) OR (Ache, Low Back[Title/Abstract])) OR (Aches, Low Back[Title/Abstract])) OR (Back Ache, Low[Title/Abstract])) OR (Back Aches, Low[Title/Abstract])) OR (Low Back Aches[Title/Abstract])) OR (Low Backache[Title/Abstract])) OR (Backache, Low[Title/Abstract])) OR (Backaches, Low[Title/Abstract])) OR (Low Backaches [Title/Abstract])) OR (Low Back Pain, Postural[Title/Abstract])) OR (Postural Low Back Pain[Title/Abstract])) OR (Low Back Pain, Posterior Compartment [Title/Abstract])) OR (Low Back Pain, Recurrent[Title/Abstract])) OR (Recurrent Low Back Pain[Title/Abstract])) OR (Low Back Pain, Mechanical [Title/Abstract])) OR (Mechanical Low Back Pain[Title/Abstract]) | 43,661 |
| **#2** | (((((((((((((((((((((((((((((((((((((((((((((((((((((((((((((((((((((((((((((((((((((((((((((((((((((((((((((((((((((((((((((((((((((((((((Sport[Title/Abstract]) OR (Athletics[Title/Abstract])) OR (Athletic[Title/Abstract])) OR (Exercises[Title/Abstract])) OR (Physical Activity [Title/Abstract])) OR (Activities, Physical[Title/Abstract])) OR (Activity, Physical [Title/Abstract])) OR (Physical Activities[Title/Abstract])) OR (Exercise, Physical [Title/Abstract])) OR (Exercises, Physical[Title/Abstract])) OR (Physical Exercise [Title/Abstract])) OR (Physical Exercises[Title/Abstract])) OR (Acute Exercise [Title/Abstract])) OR (Acute Exercises[Title/Abstract])) OR (Exercise, Acute [Title/Abstract])) OR (Exercises, Acute[Title/Abstract])) OR (Exercise, Isometric [Title/Abstract])) OR (Exercises, Isometric[Title/Abstract])) OR (Isometric Exercises [Title/Abstract])) OR (Isometric Exercise[Title/Abstract])) OR (Exercise, Aerobic [Title/Abstract])) OR (Aerobic Exercise[Title/Abstract])) OR (Aerobic Exercises [Title/Abstract])) OR (Exercises, Aerobic[Title/Abstract])) OR (Exercise Training [Title/Abstract])) OR (Exercise Trainings[Title/Abstract])) OR (Training, Exercise [Title/Abstract])) OR (Trainings, Exercise[Title/Abstract])) OR (walking [Title/Abstract])) OR (Ambulation[Title/Abstract])) OR (Training, Resistance [Title/Abstract])) OR (Strength Training[Title/Abstract])) OR (Training, Strength [Title/Abstract])) OR (Weight-Lifting Strengthening Program[Title/Abstract])) OR (Strengthening Program, Weight-Lifting[Title/Abstract])) OR (Strengthening Programs, Weight-Lifting[Title/Abstract])) OR (Weight Lifting Strengthening Program [Title/Abstract])) OR (Weight-Lifting Strengthening Programs[Title/Abstract])) OR (Weight-Lifting Exercise Program[Title/Abstract])) OR (Exercise Program, Weight-Lifting[Title/Abstract])) OR (Exercise Programs, Weight-Lifting [Title/Abstract])) OR (Weight Lifting Exercise Program[Title/Abstract])) OR (Weight-Lifting Exercise Programs[Title/Abstract])) OR (Weight-Bearing Strengthening Program[Title/Abstract])) OR (Strengthening Program, Weight-Bearing [Title/Abstract])) OR (Strengthening Programs, Weight-Bearing [Title/Abstract])) OR (Weight Bearing Strengthening Program[Title/Abstract])) OR (Weight-Bearing Strengthening Programs [Title/Abstract])) OR (Weight-Bearing Exercise Program [Title/Abstract])) OR (Exercise Program, Weight-Bearing [Title/Abstract])) OR (Exercise Programs, Weight-Bearing[Title/Abstract])) OR (Weight Bearing Exercise Program[Title/Abstract])) OR (Weight-Bearing Exercise Programs[Title/Abstract])) OR (core stability exercise[Title/Abstract])) OR (core control exercise[Title/Abstract])) OR (Training, Autogenic[Title/Abstract])) OR (Progressive Muscle Relaxation [Title/Abstract])) OR (Muscle Relaxation, Progressive[Title/Abstract])) OR (Relaxation, Progressive Muscle[Title/Abstract])) OR (Progressive Relaxation [Title/Abstract])) OR (Relaxation, Progressive[Title/Abstract])) OR (core strength exercise[Title/Abstract])) OR (sling exercise[Title/Abstract])) OR (Exercise, Muscle Stretching[Title/Abstract])) OR (Muscle Stretching Exercise[Title/Abstract])) OR (Static Stretching[Title/Abstract])) OR (Stretching, Static[Title/Abstract])) OR (Active Stretching[Title/Abstract])) OR (Stretching, Active[Title/Abstract])) OR (Static-Active Stretching[Title/Abstract])) OR (Static Active Stretching[Title/Abstract])) OR (Stretching, Static-Active[Title/Abstract])) OR (Isometric Stretching[Title/Abstract])) OR (Stretching, Isometric[Title/Abstract])) OR (Ballistic Stretching[Title/Abstract])) OR (Stretching, Ballistic[Title/Abstract])) OR (Dynamic Stretching[Title/Abstract])) OR (Stretching, Dynamic[Title/Abstract])) OR (Proprioceptive Neuromuscular Facilitation (PNF) Stretching[Title/Abstract])) OR (PNF Stretching[Title/Abstract])) OR (Stretching, PNFORPNF Stretching Exercise[Title/Abstract])) OR (Exercise, PNF Stretching[Title/Abstract])) OR (PNF Stretching Exercises[Title/Abstract])) OR (Stretching Exercise, PNF[Title/Abstract])) OR (Exercise, Breathing[Title/Abstract])) OR (Respiratory Muscle Training[Title/Abstract])) OR (Muscle Training, Respiratory[Title/Abstract])) OR (Training, Respiratory Muscle[Title/Abstract])) OR (Exergamings[Title/Abstract])) OR (Active-Video Gaming[Title/Abstract])) OR (Active Video Gaming[Title/Abstract])) OR (Active-Video Gamings[Title/Abstract])) OR (Gamings, Active-Videog, Active-Video[Title/Abstract])) OR (Virtual Reality Exercise[Title/Abstract])) OR (Exercise, Virtual Reality[Title/Abstract])) OR (Exercises, Virtual Reality[Title/Abstract])) OR (Virtual Reality Exercises [Title/Abstract])) OR (Exergames[Title/Abstract])) OR (Exergame [Title/Abstract])) OR (Tai-ji[Title/Abstract])) OR (Tai Chi[Title/Abstract])) OR (Chi, Tai[Title/Abstract])) OR (Tai Ji Quan[Title/Abstract])) OR (Ji Quan, Tai[Title/Abstract])) OR (Quan, Tai Ji[Title/Abstract])) OR (Taiji[Title/Abstract])) OR (Taijiquan[Title/Abstract])) OR (yoga[Title/Abstract])) OR (Movement Techniques, Exercise[Title/Abstract])) OR (Pilates-Based Exercises[Title/Abstract])) OR (Exercises, Pilates-Based [Title/Abstract])) OR (Pilates Based Exercises[Title/Abstract])) OR (Pilates Training [Title/Abstract])) OR (Training, Pilates[Title/Abstract])) OR (Circuit Based Exercise[Title/Abstract])) OR (Circuit-Based Exercises[Title/Abstract])) OR (Exercise, Circuit-Based[Title/Abstract])) OR (Exercises, Circuit-Based[Title/Abstract])) OR (Circuit Training[Title/Abstract])) OR (Training, Circuit[Title/Abstract])) OR (Modalities, Physical Therapy[Title/Abstract])) OR (Modality, Physical Therapy [Title/Abstract])) OR (Physical Therapy Modality[Title/Abstract])) OR (Physiotherapy (Techniques[Title/Abstract]))) OR (Physiotherapies (Techniques[Title/Abstract]))) OR (Physical[Title/Abstract])) OR (Therapy Techniques[Title/Abstract])) OR (Physical Therapy Technique[Title/Abstract])) OR (Techniques, Physical Therapy [Title/Abstract])) OR (Group Physiotherapy[Title/Abstract])) OR (Group Physiotherapies[Title/Abstract])) OR (Physiotherapies, Group[Title/Abstract])) OR (Physiotherapy, Group[Title/Abstract])) OR (Physical Therapy[Title/Abstract])) OR (Physical Therapies[Title/Abstract])) OR (Therapy, Physical[Title/Abstract])) OR (Therapy, Physical[Title/Abstract])) OR (Neurological Physiotherapy[Title/Abstract])) OR (Physiotherapy, Neurological[Title/Abstract])) OR (Neurophysiother [Title/Abstract]) | 1109630 |
| **#3** | ((randomized controlled trial[Title/Abstract]) OR (RCT [Title/Abstract])) OR (randomized[Title/Abstract]) | 642,176 |
| **#4** | (((#1) AND (#2)) AND (#3)) | 2173 |

**Web of science**

| Number | Search terms | Results |
| --- | --- | --- |
| **#1** | TI=(Back Pain, Low OR Back Pains, Low OR Low Back Pain OR Pain, Low Back OR Pains, Low Back OR Lumbago OR Lower Back Pain OR Back Pain, Lower OR Back Pains, Lower OR Lower Back Pains OR Pain, Lower Back OR Pains, Lower Back OR Low Back Ache OR Ache, Low Back OR Aches, Low Back OR Back Ache, Low OR Back Aches, Low OR Low Back Aches OR Low Backache OR Backache, Low OR backache, Low OR Low backache OR Low Back Pain, Postural OR Postural Low Back Pain OR Low Back Pain, Posterior Compartment OR Low Back Pain, Recurrent OR Recurrent Low Back Pain OR Low Back Pain, Mechanical OR Mechanical Low Back Pain ) | 69626 |
| **#2** | TI=(Sport OR Athletics OR Athletic OR Exercises OR Physical Activity OR Activities, Physical OR Activity, Physical OR Physical Activities OR Exercise, Physical OR Exercises, Physical OR Physical Exercise OR Physical Exercises OR Acute Exercise OR Acute Exercises OR Exercise, Acute OR Exercises, Acute OR Exercise, Isometric OR Exercises, Isometric OR Isometric Exercises OR Isometric Exercise OR Exercise, Aerobic OR Aerobic Exercise OR Aerobic Exercises OR Exercises, Aerobic OR Exercise Training OR Exercise Trainings OR Training, Exercise OR Trainings, Exercise OR walking OR Ambulation OR Training, Resistance OR Strength Training OR Training, Strength OR Weight-Lifting Strengthening Program OR Strengthening Program, Weight-Lifting OR Strengthening Programs, Weight-Lifting OR Weight Lifting Strengthening Program OR Weight-Lifting Strengthening Programs OR Weight-Lifting Exercise Program OR Exercise Program, Weight-Lifting OR Exercise Programs, Weight-Lifting OR Weight Lifting Exercise Program OR Weight-Lifting Exercise Programs OR Weight-Bearing Strengthening Program OR Strengthening Program, Weight-Bearing OR Strengthening Programs, Weight-Bearing OR Weight Bearing Strengthening Program OR Weight-Bearing Strengthening Programs OR Weight-Bearing Exercise Program OR Exercise Program, Weight-Bearing OR Exercise Programs, Weight-Bearing OR Weight Bearing Exercise Program OR Weight-Bearing Exercise Programs OR core stability exercise OR core control exercise OR Training, Autogenic OR Progressive Muscle Relaxation OR Muscle Relaxation, Progressive OR Relaxation, Progressive Muscle OR Progressive Relaxation OR Relaxation, Progressive OR core strength exercise OR sling exercise OR Exercise, Muscle Stretching OR Muscle Stretching Exercise OR Static Stretching OR Stretching, Static OR Active Stretching OR Stretching, Active OR Static-Active Stretching OR Static Active Stretching OR Stretching, Static-Active OR Isometric Stretching OR Stretching, Isometric OR Ballistic Stretching OR Stretching, Ballistic OR Dynamic Stretching OR Stretching, Dynamic OR Proprioceptive Neuromuscular Facilitation (PNF) Stretching OR PNF Stretching OR PNF Stretchings OR Stretching, PNFORPNF Stretching Exercise OR Exercise, PNF Stretching OR PNF Stretching Exercises OR Stretching Exercise, PNF OR Exercise, Breathing OR Respiratory Muscle Training OR Muscle Training, Respiratory OR Training, Respiratory Muscle OR Exergamings OR Active-Video Gaming OR Active Video Gaming OR Active-Video Gamings OR Gaming, Active-Video OR Gamings, Active-Video OR Virtual Reality Exercise OR Exercise, Virtual Reality OR Exercises, Virtual Reality OR Virtual Reality Exercises OR Exergames OR Exergame OR Tai-ji OR Tai Chi OR Chi, Tai OR Tai Ji Quan OR Ji Quan, Tai OR Quan, Tai Ji OR Taiji OR Taijiquan OR T'ai Chi OR Tai Chi Chuan OR yoga OR Movement Techniques, Exercise OR Exercise Movement Technics OR Pilates-Based Exercises OR Exercises, Pilates-Based OR Pilates Based Exercises OR Pilates Training OR Training, Pilates OR Circuit Based Exercise OR Circuit-Based Exercises OR Exercise, Circuit-Based OR Exercises, Circuit-Based OR Circuit Training OR Training, Circuit OR Modalities, Physical Therapy OR Modality, Physical Therapy OR Physical Therapy Modality OR Physiotherapy (Techniques) OR Physiotherapies (Techniques)OR Physical OR Therapy Techniques OR Physical Therapy Technique OR Techniques, Physical Therapy OR Group Physiotherapy OR Group Physiotherapies OR Physiotherapies, Group OR Physiotherapy, Group OR Physical Therapy OR Physical Therapies OR Therapy, Physical OR Neurological Physiotherapy OR Physiotherapy, Neurological OR Neurophysiotherap) | 717626 |
| **#3** | TI= (randomized controlled trial OR RCT OR randomized) | 341702 |
| **#4** | (((#1) AND (#2)) AND (#3)) | 500 |

**Embase:**

| Number | Search terms | Results |
| --- | --- | --- |
| **#1** | 'back pain, low'/exp OR 'back pain, low' OR (('back'/exp OR back) AND ('pain,'/exp OR pain,) AND low) OR 'back pains, low' OR (('back'/exp OR back) AND pains, AND low) OR 'low back pain'/exp OR 'low back pain' OR (low AND ('back'/exp OR back) AND ('pain'/exp OR pain)) OR 'pain, low back'/exp OR 'pain, low back' OR (('pain,'/exp OR pain,) AND low AND ('back'/exp OR back)) OR 'pains, low back' OR (pains, AND low AND ('back'/exp OR back)) OR 'lumbago'/exp OR lumbago OR 'lower back pain'/exp OR 'lower back pain' OR (lower AND ('back'/exp OR back) AND ('pain'/exp OR pain)) OR 'back pain, lower' OR (('back'/exp OR back) AND ('pain,'/exp OR pain,) AND lower) OR 'back pains, lower' OR (('back'/exp OR back) AND pains, AND lower) OR 'lower back pains' OR (lower AND ('back'/exp OR back) AND pains) OR 'pain, lower back' OR (('pain,'/exp OR pain,) AND lower AND ('back'/exp OR back)) OR 'pains, lower back' OR (pains, AND lower AND ('back'/exp OR back)) OR 'low back ache' OR (low AND ('back'/exp OR back) AND ache) OR 'ache, low back' OR (ache, AND low AND ('back'/exp OR back)) OR 'aches, low back' OR (aches, AND low AND ('back'/exp OR back)) OR 'back ache, low' OR (('back'/exp OR back) AND ache, AND low) OR 'back aches, low' OR (('back'/exp OR back) AND aches, AND low) OR 'low back aches' OR (low AND ('back'/exp OR back) AND aches) OR 'backache, low' OR (('backache,'/exp OR backache,) AND low) OR 'low backache'/exp OR 'low backache' OR (low AND ('backache'/exp OR backache)) OR 'low back pain, postural' OR (low AND ('back'/exp OR back) AND ('pain,'/exp OR pain,) AND postural) OR 'postural low back pain' OR (postural AND low AND ('back'/exp OR back) AND ('pain'/exp OR pain)) OR 'low back pain, posterior compartment' OR (low AND ('back'/exp OR back) AND ('pain,'/exp OR pain,) AND posterior AND compartment) OR 'low back pain, recurrent' OR (low AND ('back'/exp OR back) AND ('pain,'/exp OR pain,) AND recurrent) OR 'recurrent low back pain' OR (recurrent AND low AND ('back'/exp OR back) AND ('pain'/exp OR pain)) OR 'low back pain, mechanical' OR (low AND ('back'/exp OR back) AND ('pain,'/exp OR pain,) AND mechanical) OR 'mechanical low back pain'/exp OR 'mechanical low back pain' OR (mechanical AND low AND ('back'/exp OR back) AND ('pain'/exp OR pain)) | 110123 |
| **#2** | 'sport'/exp OR sport OR 'athletics'/exp OR athletics OR athletic OR exercises OR 'physical activity'/exp OR 'physical activity' OR (physical AND ('activity'/exp OR activity)) OR 'activities, physical' OR (activities, AND physical) OR 'activity, physical'/exp OR 'activity, physical' OR (activity, AND physical) OR 'physical activities' OR (physical AND activities) OR 'exercise, physical' OR (('exercise,'/exp OR exercise,) AND physical) OR 'exercises, physical' OR (exercises, AND physical) OR 'physical exercise'/exp OR 'physical exercise' OR (physical AND ('exercise'/exp OR exercise)) OR 'physical exercises' OR (physical AND exercises) OR 'acute exercise'/exp OR 'acute exercise' OR (acute AND ('exercise'/exp OR exercise)) OR 'acute exercises' OR (acute AND exercises) OR 'exercise, acute' OR (('exercise,'/exp OR exercise,) AND acute) OR 'exercises, acute' OR (exercises, AND acute) OR 'exercise, isometric'/exp OR 'exercise, isometric' OR (('exercise,'/exp OR exercise,) AND isometric) OR 'exercises, isometric' OR (exercises, AND isometric) OR 'isometric exercises' OR (isometric AND exercises) OR 'isometric exercise'/exp OR 'isometric exercise' OR (isometric AND ('exercise'/exp OR exercise)) OR 'exercise, aerobic'/exp OR 'exercise, aerobic' OR (('exercise,'/exp OR exercise,) AND aerobic) OR 'aerobic exercise'/exp OR 'aerobic exercise' OR (aerobic AND ('exercise'/exp OR exercise)) OR 'aerobic exercises' OR (aerobic AND exercises) OR 'exercises, aerobic' OR (exercises, AND aerobic) OR 'exercise training'/exp OR 'exercise training' OR (('exercise'/exp OR exercise) AND ('training'/exp OR training)) OR 'exercise trainings' OR (('exercise'/exp OR exercise) AND trainings) OR 'training, exercise' OR (('training,'/exp OR training,) AND ('exercise'/exp OR exercise)) OR 'trainings, exercise' OR (trainings, AND ('exercise'/exp OR exercise)) OR 'walking'/exp OR walking OR 'ambulation'/exp OR ambulation OR 'training, resistance' OR (('training,'/exp OR training,) AND ('resistance'/exp OR resistance)) OR 'strength training'/exp OR 'strength training' OR (('strength'/exp OR strength) AND ('training'/exp OR training)) OR 'training, strength' OR (('training,'/exp OR training,) AND ('strength'/exp OR strength)) OR 'weight-lifting strengthening program' OR (('weight lifting'/exp OR 'weight lifting') AND strengthening AND ('program'/exp OR program)) OR 'strengthening program, weight-lifting' OR (strengthening AND program, AND ('weight lifting'/exp OR 'weight lifting')) OR 'strengthening programs, weight-lifting' OR (strengthening AND programs, AND ('weight lifting'/exp OR 'weight lifting')) OR 'weight lifting strengthening program' OR (('weight'/exp OR weight) AND ('lifting'/exp OR lifting) AND strengthening AND ('program'/exp OR program)) OR 'weight-lifting strengthening programs' OR (('weight lifting'/exp OR 'weight lifting') AND strengthening AND ('programs'/exp OR programs)) OR 'weight-lifting exercise program' OR (('weight lifting'/exp OR 'weight lifting') AND ('exercise'/exp OR exercise) AND ('program'/exp OR program)) OR 'exercise program, weight-lifting' OR (('exercise'/exp OR exercise) AND program, AND ('weight lifting'/exp OR 'weight lifting')) OR 'exercise programs, weight-lifting' OR (('exercise'/exp OR exercise) AND programs, AND ('weight lifting'/exp OR 'weight lifting')) OR 'weight lifting exercise program' OR (('weight'/exp OR weight) AND ('lifting'/exp OR lifting) AND ('exercise'/exp OR exercise) AND ('program'/exp OR program)) OR 'weight-lifting exercise programs' OR (('weight lifting'/exp OR 'weight lifting') AND ('exercise'/exp OR exercise) AND ('programs'/exp OR programs)) OR 'weight-bearing strengthening program' OR (('weight bearing'/exp OR 'weight bearing') AND strengthening AND ('program'/exp OR program)) OR 'strengthening program, weight-bearing' OR (strengthening AND program, AND ('weight bearing'/exp OR 'weight bearing')) OR 'strengthening programs, weight-bearing' OR (strengthening AND programs, AND ('weight bearing'/exp OR 'weight bearing')) OR 'weight bearing strengthening program' OR (('weight'/exp OR weight) AND ('bearing'/exp OR bearing) AND strengthening AND ('program'/exp OR program)) OR 'weight-bearing strengthening programs' OR (('weight bearing'/exp OR 'weight bearing') AND strengthening AND ('programs'/exp OR programs)) OR 'weight-bearing exercise program' OR (('weight bearing'/exp OR 'weight bearing') AND ('exercise'/exp OR exercise) AND ('program'/exp OR program)) OR 'exercise program, weight-bearing' OR (('exercise'/exp OR exercise) AND program, AND ('weight bearing'/exp OR 'weight bearing')) OR 'exercise programs, weight-bearing' OR (('exercise'/exp OR exercise) AND programs, AND ('weight bearing'/exp OR 'weight bearing')) | 1,435,087 |
| **#3** | 'weight bearing exercise program' OR (('weight'/exp OR weight) AND ('bearing'/exp OR bearing) AND ('exercise'/exp OR exercise) AND ('program'/exp OR program)) OR 'weight-bearing exercise programs' OR (('weight bearing'/exp OR 'weight bearing') AND ('exercise'/exp OR exercise) AND ('programs'/exp OR programs)) OR 'core stability exercise'/exp OR 'core stability exercise' OR (('core'/exp OR core) AND ('stability'/exp OR stability) AND ('exercise'/exp OR exercise)) OR 'core control exercise' OR (('core'/exp OR core) AND ('control'/exp OR control) AND ('exercise'/exp OR exercise)) OR 'training, autogenic' OR (('training,'/exp OR training,) AND autogenic) OR 'progressive muscle relaxation'/exp OR 'progressive muscle relaxation' OR (progressive AND ('muscle'/exp OR muscle) AND ('relaxation'/exp OR relaxation)) OR 'muscle relaxation, progressive' OR (('muscle'/exp OR muscle) AND ('relaxation,'/exp OR relaxation,) AND progressive) OR 'relaxation, progressive muscle' OR (('relaxation,'/exp OR relaxation,) AND progressive AND ('muscle'/exp OR muscle)) OR 'progressive relaxation' OR (progressive AND ('relaxation'/exp OR relaxation)) OR 'relaxation, progressive' OR (('relaxation,'/exp OR relaxation,) AND progressive) OR 'core strength exercise' OR (('core'/exp OR core) AND ('strength'/exp OR strength) AND ('exercise'/exp OR exercise)) OR 'sling exercise'/exp OR 'sling exercise' OR (('sling'/exp OR sling) AND ('exercise'/exp OR exercise)) OR 'exercise, muscle stretching' OR (('exercise,'/exp OR exercise,) AND ('muscle'/exp OR muscle) AND ('stretching'/exp OR stretching)) OR 'muscle stretching exercise' OR (('muscle'/exp OR muscle) AND ('stretching'/exp OR stretching) AND ('exercise'/exp OR exercise)) OR 'static stretching'/exp OR 'static stretching' OR (static AND ('stretching'/exp OR stretching)) OR 'stretching, static' OR (('stretching,'/exp OR stretching,) AND static) OR 'active stretching' OR (active AND ('stretching'/exp OR stretching)) OR 'stretching, active' OR (('stretching,'/exp OR stretching,) AND active) OR 'static-active stretching' OR ('static active' AND ('stretching'/exp OR stretching)) OR 'static active stretching' OR (static AND active AND ('stretching'/exp OR stretching)) OR 'stretching, static-active' OR (('stretching,'/exp OR stretching,) AND 'static active') | 210213 |
| **#4** | 'isometric stretching' OR (isometric AND ('stretching'/exp OR stretching)) OR 'stretching, isometric' OR (('stretching,'/exp OR stretching,) AND isometric) OR 'ballistic stretching' OR (ballistic AND ('stretching'/exp OR stretching)) OR 'stretching, ballistic' OR (('stretching,'/exp OR stretching,) AND ballistic) OR 'dynamic stretching' OR (('dynamic'/exp OR dynamic) AND ('stretching'/exp OR stretching)) OR 'stretching, dynamic' OR (('stretching,'/exp OR stretching,) AND ('dynamic'/exp OR dynamic)) OR 'proprioceptive neuromuscular facilitation stretching' OR (proprioceptive AND neuromuscular AND ('facilitation'/exp OR facilitation) AND pnf AND ('stretching'/exp OR stretching)) OR 'pnf stretching' OR (pnf AND ('stretching'/exp OR stretching)) OR 'pnf stretchings' OR (pnf AND stretchings) OR 'stretching, pnforpnf stretching exercise' OR (('stretching,'/exp OR stretching,) AND pnforpnf AND ('stretching'/exp OR stretching) AND ('exercise'/exp OR exercise)) OR 'exercise, pnf stretching' OR (('exercise,'/exp OR exercise,) AND pnf AND ('stretching'/exp OR stretching)) OR 'pnf stretching exercises' OR (pnf AND ('stretching'/exp OR stretching) AND exercises) OR 'stretching exercise, pnf' OR (('stretching'/exp OR stretching) AND ('exercise,'/exp OR exercise,) AND pnf) OR 'exercise, breathing'/exp OR 'exercise, breathing' OR (('exercise,'/exp OR exercise,) AND ('breathing'/exp OR breathing)) OR 'respiratory muscle training'/exp OR 'respiratory muscle training' OR (('respiratory'/exp OR respiratory) AND ('muscle'/exp OR muscle) AND ('training'/exp OR training)) OR 'muscle training, respiratory' OR (('muscle'/exp OR muscle) AND ('training,'/exp OR training,) AND ('respiratory'/exp OR respiratory)) OR 'training, respiratory muscle' OR (('training,'/exp OR training,) AND ('respiratory'/exp OR respiratory) AND ('muscle'/exp OR muscle)) OR exergamings OR 'active-video gaming'/exp OR 'active-video gaming' OR ('active video' AND gaming) OR 'active video gaming'/exp OR 'active video gaming' OR (active AND ('video'/exp OR video) AND gaming) OR 'active-video gamings' OR ('active video' AND gamings) OR 'gaming, active-video' OR (gaming, AND 'active video') OR 'gamings, active-video' OR (gamings, AND 'active video') OR 'virtual reality exercise'/exp OR 'virtual reality exercise' OR (virtual AND ('reality'/exp OR reality) AND ('exercise'/exp OR exercise)) OR 'exercise, virtual reality' OR (('exercise,'/exp OR exercise,) AND virtual AND ('reality'/exp OR reality)) OR 'exercises, virtual reality' OR (exercises, AND virtual AND ('reality'/exp OR reality)) OR 'virtual reality exercises' OR (virtual AND ('reality'/exp OR reality) AND exercises) OR exergames OR 'exergame'/exp OR exergame OR 'tai chi'/exp OR 'tai chi' OR (tai AND chi) OR 'chi, tai' OR (chi, AND tai) OR 'tai chi chuan'/exp OR 'tai chi chuan' OR (tai AND chi AND chuan) OR 'yoga'/exp OR yoga OR 'movement techniques, exercise' OR (('movement'/exp OR movement) AND techniques, AND ('exercise'/exp OR exercise)) OR 'exercise movement technics' OR (('exercise'/exp OR exercise) AND ('movement'/exp OR movement) AND technics) OR 'pilates-based exercises' OR ('pilates based' AND exercises) OR 'pilates based exercises' OR (('pilates'/exp OR pilates) AND based AND exercises) OR 'pilates training' OR (('pilates'/exp OR pilates) AND ('training'/exp OR training)) OR 'training, pilates' OR (('training,'/exp OR training,) AND ('pilates'/exp OR pilates)) OR 'circuit based exercise'/exp OR 'circuit based exercise' OR (('circuit'/exp OR circuit) AND based AND ('exercise'/exp OR exercise)) OR 'circuit-based exercises' OR ('circuit based' AND exercises) OR 'exercise, circuit-based' OR (('exercise,'/exp OR exercise,) AND 'circuit based') OR 'exercises, circuit-based' OR (exercises, AND 'circuit based') OR 'circuit training'/exp OR 'circuit training' OR (('circuit'/exp OR circuit) AND ('training'/exp OR training)) OR 'training, circuit' OR (('training,'/exp OR training,) AND ('circuit'/exp OR circuit)) OR 'modalities, physical therapy' OR (modalities, AND physical AND ('therapy'/exp OR therapy)) OR 'modality, physical therapy' OR (modality, AND physical AND ('therapy'/exp OR therapy)) OR 'physical therapy modality' OR (physical AND ('therapy'/exp OR therapy) AND ('modality'/exp OR modality)) | 92860 |
| **#5** | #2 OR #3 OR #4 | 1,470,119 |
| **#6** | randomized AND controlled AND ('trial'/exp OR trial) OR rct OR 'randomized controlled trial'/exp OR 'randomized controlled trial' | 108475 |
| **#7** | #1 AND #5 AND #6 | 3459 |

**Cochrane Library:**

| Number | Search terms | Results |
| --- | --- | --- |
| **#1** | Back Pain, Low OR Back Pains, Low OR Low Back Pain OR Pain, Low Back OR Pains, Low Back OR Lumbago OR Lower Back Pain OR Back Pain, Lower OR Back Pains, Lower OR Lower Back Pains OR Pain, Lower Back OR Pains, Lower Back OR Low Back Ache OR Ache, Low Back OR Aches, Low Back OR Back Ache, Low OR Back Aches, Low OR Low Back Aches OR Low Backache OR Backache, Low OR backache, Low OR Low backache OR Low Back Pain, Postural OR Postural Low Back Pain OR Low Back Pain, Posterior Compartment OR Low Back Pain, Recurrent OR Recurrent Low Back Pain OR Low Back Pain, Mechanical OR Mechanical Low Back Pain | 17647 |
| **#2** | Sport OR Athletics OR Athletic OR Exercises OR Physical Activity OR Activities, Physical OR Activity, Physical OR Physical Activities OR Exercise, Physical OR Exercises, Physical OR Physical Exercise OR Physical Exercises OR Acute Exercise OR Acute Exercises OR Exercise, Acute OR Exercises, Acute OR Exercise, Isometric OR Exercises, Isometric OR Isometric Exercises OR Isometric Exercise OR Exercise, Aerobic OR Aerobic Exercise OR Aerobic Exercises OR Exercises, Aerobic OR Exercise Training OR Exercise Trainings OR Training, Exercise OR Trainings, Exercise OR walking OR Ambulation OR Training, Resistance OR Strength Training OR Training, Strength OR Weight-Lifting Strengthening Program OR Strengthening Program, Weight-Lifting OR Strengthening Programs, Weight-Lifting OR Weight Lifting Strengthening Program OR Weight-Lifting Strengthening Programs OR Weight-Lifting Exercise Program OR Exercise Program, Weight-Lifting OR Exercise Programs, Weight-Lifting OR Weight Lifting Exercise Program OR Weight-Lifting Exercise Programs OR Weight-Bearing Strengthening Program OR Strengthening Program, Weight-Bearing OR Strengthening Programs, Weight-Bearing OR Weight Bearing Strengthening Program OR Weight-Bearing Strengthening Programs OR Weight-Bearing Exercise Program OR Exercise Program, Weight-Bearing OR Exercise Programs, Weight-Bearing | 158351 |
| **#3** | Weight Bearing Exercise Program OR Weight-Bearing Exercise Programs OR core stability exercise OR core control exercise OR Training, Autogenic OR Progressive Muscle Relaxation OR Muscle Relaxation, Progressive OR Relaxation, Progressive Muscle OR Progressive Relaxation OR Relaxation, Progressive OR core strength exercise OR sling exercise OR Exercise, Muscle Stretching OR Muscle Stretching Exercise OR Static Stretching OR Stretching, Static OR Active Stretching OR Stretching, Active OR Static-Active Stretching OR Static Active Stretching OR Stretching, Static-Active OR Isometric Stretching OR Stretching, Isometric OR Ballistic Stretching OR Stretching, Ballistic OR Dynamic Stretching OR Stretching, Dynamic OR Proprioceptive Neuromuscular Facilitation (PNF) Stretching OR PNF Stretching OR PNF Stretchings OR Stretching, PNFORPNF Stretching Exercise OR Exercise, PNF Stretching OR PNF Stretching Exercises OR Stretching Exercise, PNF OR Exercise, Breathing OR Respiratory Muscle Training OR Muscle Training, Respiratory OR Training, Respiratory Muscle OR Exergamings OR Active-Video Gaming OR Active Video Gaming OR Active-Video Gamings OR Gaming, Active-Video OR Gamings, Active-Video OR Virtual Reality Exercise OR Exercise, Virtual Reality OR Exercises, Virtual Reality OR Virtual Reality Exercises OR Exergames OR Exergame OR Tai-ji OR Tai Chi OR Chi, Tai OR Tai Ji Quan OR Ji Quan, Tai OR Quan, Tai Ji OR Taiji OR Taijiquan OR T'ai Chi OR Tai Chi Chuan OR yoga OR Movement Techniques, Exercise OR Exercise Movement Technics OR Pilates-Based Exercises OR Exercises, Pilates-Based OR Pilates Based Exercises OR Pilates Training OR Training, Pilates OR Circuit Based Exercise OR Circuit-Based Exercises OR Exercise, Circuit-Based OR Exercises, Circuit-Based OR Circuit Training OR Training, Circuit OR Modalities, Physical Therapy OR Modality, Physical Therapy OR Physical Therapy Modality OR Physiotherapy (Techniques) OR Physiotherapies (Techniques)OR Physical OR Therapy Techniques OR Physical Therapy Technique OR Techniques, Physical Therapy OR Group Physiotherapy OR Group Physiotherapies OR Physiotherapies, Group OR Physiotherapy, Group OR Physical Therapy OR Physical Therapies OR Therapy, Physical OR Neurological Physiotherapy OR Physiotherapy, Neurological OR Neurophysiotherap | 199468 |
| **#4** | #2 OR #3 | 250228 |
| **#5** | randomized controlled trial OR RCT OR randomized | 1131834 |
| **#6** | #1 AND #4 AND #5 | 6529 |
