# Supplementary material for: Exercise intervention for patients with chronic low back pain: a systematic review and network meta-analysis
Source: Front Public Health. 2023 Nov 17;11:1155225. doi: 10.3389/fpubh.2023.1155225 (PMC10687566; doi:10.3389/fpubh.2023.1155225)
Supplement: Supplementary file 1 [file Data_Sheet_1.zip › Supplementary Appendix 4.DOCX]

| Study | Country | Sample size  (M/F) | Age (years M/F) | Sample size | Exercise/Control | Height | Weight | Intervention time | Outcome |
| --- | --- | --- | --- | --- | --- | --- | --- | --- | --- |
| Hosseinifar 2013 | Iran | NA | CSE:40.1±10.8 MKE:36.6±8.2 | CSE:18  MKE:19 | CSE/MKE | CSE:170.5±8.5  MKE:172.1±8 | CSE:75±13 MKE:78.4±10.6 | 3 times/week, 6 weeks | VAS |
| Liu 2019 | China | 11/32 | TC:58.13±5.38 CSE:58.4±5.08 NIP:60.67±2.58 | TC:15  CSE:15  NIP:13 | TC/CSE/NIP | TC:159.53±7.24  CSE:162.53±8.21  NIP:159.00±7.17 | TC:58.93±9.93 CSE:63.33±9.08 NIP:63.47±12.05 | 3 times/week, 12 weeks | VAS |
| Suh 2019 | Korea | 11/  25 | AE:54.15±13.89 CSE:57.40±15.88 MUE:54.75±14.98 | AE:15  CSE:15  MUE:15 | AE/CSE/ MUE | NA | NA | 60minutes/time,5 times/week, 6 weeks | VAS |
| Cho 2014 | Korea | 11/19 | CSE:38.1±7.9 COR:36.5±7.7 | CSE:15  COR:15 | CSE/COR | CSE:165.2±7.6 COR:164.6±8.2 | CSE:66.5±11.5 COR:65.1±10.7 | 3 times/week, 4weeks | VAS |
| Kim 2014 | Korea | 0/30 | MUE:44.33  CSE:50.46 | MUE:15  CSE:15 | MUE/CSE | MUE:158.33 CSE:156.55 | MUE:58.15 CSE:56.18 | 3 times/week, 4 weeks | ODI; VAS |
| Kankaanpäă 1999 | Finland | 35/19 | CSE: 40.7±8.6/ 38.9±8.2 MAT:38.0±6.9/40.6±8.1 | CSE:30  MAT:24 | CSE/ MAT | CSE:177.0±5.2/165.0±10.3  MAT:175.9±4.9/167.3±3.8 | CSE:82.3±9.7/ 70.6±16.2 MAT:75.8±8.4/71.9±10.0 | 12 weeks | VAS |
| Goran 2018 | Croatia | 16/14 | 34.2 | Yoga:15  ED:15 | Yoga/ED | NA | NA | 2 times/week, 8weeks | NRS; ODI |
| Koldaş 2008 | Turkey | 8/29 | MUE:37.1±6.5 HE:42.1±9.5 | MUE:19  HE:19 | MUE/HE | NA | NA | 3 times/week, 6 weeks | RMDQ |
| Cho 2015 | Korea | 9/21 | CSE:48.1±6.9 PT:44.0±6.7 | CSE:15  PT:15 | CSE/PT | CSE:160.8±6.3 PT:163.6±8.2 | CSE:61.9±9.3 PT:60.5±12.2 | 3 times/week, 6 weeks | ODI |
| Oh 2015 | Korea | NA | CSE:46.0±3.37 SE:46.2±3.22 NIP:44.2±2.70 | CSE:10  SE:10  NIP:10 | CSE/SEG/NIP | CSE:172.0±3.20 SE:170.1±4.48 NIP:169.9±4.88 | CSE:69.3±9.18 SE:71.0±10.47 NIP:69.2±7.79 | 30 minutes/time, 5 times/week, 12 weeks | ODI; VAS |
| Kang 2016 | Korea | NA | OE:42.5±5.3 CSE:40.1±5.3 | OE:10  CSE:10 | OE/CSE | OE:168.5±5.7 CSE:166.3±4.9 | OE:64.3±9.8 CSE:65.4±8.2 | 4 times/week, 6 weeks | ODI |
| Mannion 2001 | Finland | 64/84 | PT:46.3±10.1 AE:45.2±9.7 MUT:43.7±10.1 | PT:49  AE:49  MUT:50 | PT/AE/MUT | PT:171±9 AE:170±11 MUT:172±9 | PT:71.4±11.0 AE:68.0±12.3 MUT：70.3±13.4 | 6 months | VAS; RMDQ |
| Ronald 2005 | USA | 15/14 | CSE:54±15 MKE:44±16 | CSE:15  MKE:15 | CSE/MKE | NA | NA | 6 weeks | VAS |
| Akbari 2008 | Iran | NA | MCE:39.6±3.5 COR:40±3.6 | MCE:25  COR:24 | MCE/COR | MCE:171.2±2.7 COR:172.08±2.2 | MCE:73.7±3.1 COR:74.6±2.4 | 30 minutes/time, 2 times/week, 8 weeks | VAS |
| Moon 2013 | Korea | 14/7 | CSE:28.4±5.0 MUT:28.6±4.9 | CSE:11  MUT:10 | CSE/MUT | CSE:171.4±5.1 MUT:172.3±6.3 | CSE:67.4±12.9 MUT:68.2±14.3 | 60 minutes/time, 2 times/week, 8 weeks | ODI; VAS |
| Bronfort 2011 | USA | 119/182 | HE:45.6±10.3 MAT:45.2±10.8 MUE:44.5±11.8 | HE:101  MAT:100  MUE:100 | HE/MAT/MUE | HE:169.1±10.1 MAT:169.0±10.3 MUE:168.6±9.9 | HE:80.3±15.6 MAT:78.9±17.8 MUE:79.9±17.9 | 6 weeks | ODI; NRS |
| Cai 2017 | Singapore | NA | 27.3±5.5 | MUT:28  CSE:28  COR:28 | MUT/CSE/COR | NA | 61.2± 11.8 | 8 weeks | NRS |
| Demirel 2019 | Turkey | 15/62 | Yoga:44.25±8.71 CSE:45.59±12.32 | Yoga:40  CSE:40 | Yoga/CSE | NA | NA | 3 times/week, 6 weeks | ODI; VAS |
| Dundar 2009 | Turkey | 34/31 | WPA:35.3±7.8 HE:34.8±8.3 | WPA:32  HE:33 | WPA/HE | NA | NA | 60 minutes/time, 7 times/week, 4 weeks | VAS |
| Roh 2016 | Korea | NA | SE:49.5±10.6 PT:50.5±9.1 | SE:53  PT:49 | SE/PT | SE;165.9±8.6 PT:164.5±6.3 | SE:64.9±13.6 PT:61.6±7.0 | 3 times/week, 12 weeks | VAS |
| Rydeard 2006 | Canada | 14/25 | PE:37±9  NIP:34±8 | PE:21  NIP:18 | PE/NIP | PE:169±8  NIP:171±10 | PE:68±14 NIP:69±15 | 4 weeks | NRS; ODI |
| Garcia 2013 | Brazil | 39/109 | MKE:53.70±1.53 OE:54.16±1.57 | MKE:74  OE:74 | MKE/OE | MKE:161±1  OE:164±1 | MKE:71.70±1.59 OE:73.73±1.59 | 1 times/week, 4 weeks | NRS; ODI; |
| Torstensen 1998 | Norway | NA | PT:43.0±12.0 AE:39.9±11.4 | PT:67  AE:70 | PT/AE | PT:175.0±8.9 AE:174.0±11.4 | PT:77.7±18.5 AE:78.7±17.0 | 3 times/week, 12 weeks | ODI; VAS |
| Mannion 1999 | Finland. | 53/84 | PT:46.3±10.1 MUT:43.7±10.1 AE:45.2±9.7 | PT:49  MUT:49  AE:50 | PT/MUT/AE | PT:171±9  MUT:172±9  AE:170±11 | PT:71.4±11 MUT:70.3±13.4 AE:68.0±12.3 | 2 times/week, 3 months | VAS; RMDQ |
| Macedo 2012 | Canada | 70/102 | MCE:48.7±13.7 OE:49.6±16.3 | MCE:86  OE:86 | MCE/OE | MCE:166.9±9.2  OE:168.5±10.1 | MCE:75.7±19.3 OE:80.8±16.2 | 12 weeks | NRS; ODI |
| Lee 2016 | Korea | NA | MUT:42.7±13.4 MUE:46.7±8.1 NIP:43.3±9.9 | MUT:15  MUE:15  NIP:6 | MUT/MUE/NIP | MUT:169.0±8.3 MUE:168.1±9.3 NIP:169.3±9.2 | MUT:68.8±11.8 MUE:67.6±10.2 NIP:80.1±19.9 | 2 times/week, 12 weeks | VAS; RMDQ |
| Kell 2009 | Canada | 11/7 | MUT:40.1±8.7 AE:36.7±8.9 | MUT:9  AE:9 | MUT/AE | MUT:174±8 AE:173±10 | NA | 3 times/week, 8 weeks | ODI; VAS |
| Chatzitheodorou 2007 | Greece | 11/9 | AE:42.4±12.7 MAT:41.5±12.9 | AE:10  MAT:10 | AE/MAT | NA | NA | 12 weeks | RMDQ |
| Murtezani 2015 | Kosovo | 125/95 | MKE:48.8±8.9 PT:47.5±8.8 | MKE:111  PT:109 | MKE/PT | MKE:174.0±7.1 PT:173.7±7.0 | MKE:77.8±10.9 PT:78.5±10.2 | 4 weeks | ODI; VAS |
| Unsgaard 2010 | Norway | 33/76 | MCE:40.9±11.5 SE:43.4±10.2 OE:36.0±10.3 | MCE:36  SE:36  OE:37 | MCE/SE/OE | MCE:171.4±7.7 SE:172.6±7.7 OE:171.2±8.7 | MCE:73.3±11.6 SE:74.5±11.6 OE:71.1±9.9 | 1 times/week, 8 weeks | NRS; ODI |
| Murtezani 2011 | Kosovo | 52/49 | AE:50±9.6 PT:51±10 | AE:50  PT:51 | AE/ PT | AE:174.3±6.9 PT:173.6±6.2 | AE:77.5±9.2 PT:78.5±11.6 | 12 weeks | ODI; VAS |
| Szulc 2015 | Poland | NA | 44 | MKE:20  MUE:20 | MKE/MUE | NA | NA | 10 days | ODI; VAS |
| Tekur 2012 | India | 44/36 | Yoga:49±3.6  PT:48±4 | Yoga:40  PT:40 | Yoga/ PT | NA | NA | 7 days | VAS |
| Tekur 2008 | India | 44/36 | Yoga:49±3.6 MUT:48±4 | Yoga:45  MUT:46 | Yoga/MUT | NA | NA | 7 days | ODI |
| Sipaviciene 2020 | Lithuania. | NA | CSE:38.3±5.1 MUT:38.5±6.2 | CSE:35  MUT:35 | CSE/MUT | CSE:168.3±3.7 MUT:167.8±4.7 | CSE:65.1±7.9 MUT:66.3±8.2 | 2 times/week, 20 weeks | ODI; VAS |
| Shnayderman 2013 | Israel | 11/41 | AE:47.0±10 MUT:43.6±13.5 | AE:26  MUT:26 | AE/ MUT | AE:161±9 MUT:162±8 | AE:75.8±16.5 MUT:71.9±12.6 | 2 times/week, 6 weeks | ODI |
| Shamsi 2016 | Iran | 13/30 | CSE:39.2±11.7 COR:47.9±10.2 | CSE:24  COR:24 | CSE/COR | CSE:166.4±9.1 COR:163.7±8.1 | CSE:70.1±15.1 COR:74.3±10.5 | 3 times/week, 6 weeks | ODI; VAS |
| Shamsi 2015 | Iran | 12/27 | CSE:38.5±11.9 MUT:47.7±10.4 | CSE:19  MUT:20 | CSE/ MUT | CSE:166.7±8.6 MUT:163.7±8.3 | CSE:68.9±15.7 MUT:73.1±8.9 | 3 times/week, 6 weeks | ODI; VAS |
| Waseem 2019 | Pakistan | 71/37 | CSE:46.39±7.43 COR:45.50±6.61 | CSE:53  COR:55 | CSE/COR | CSE:162±8 COR:160±8 | CSE:64.03±10 COR:63.69±9.15 | 6 weeks | ODI |
| Valenza 2017 | Spain | 13/41 | PE:38±12 NIP:40±16 | PE:27  NIP:27 | PE/NIP | NA | NA | 2 times/week, 8 weeks | ODI; VAS |
| Yozbatiran 2004 | Turkey | 7/23 | OE:38.6±6.57 WPA:39.60±6.33 | OE:5  WPA:15 | OE/WPA | NA | NA | 3 times/week, 4 weeks | NRS; ODI |
| Williams 2005 | USA | 14/30 | Yoga:48.7±10.6 ED:48.0±1.96 | Yoga:30  ED:30 | Yoga/ED | NA | NA | 16 weeks | VAS |
| Mohamed 2012 | Egypt | NA | PE:23.45±2.4 NIP:26.22 ±3.6 | PE:10  NIP:10 | PE/NIP | PE:171.05±5.5 NIP:168.32±7.3 | PE:70.76±8.4 NIP:68.11±11.2 | 4 times/week, 8 weeks | RMDQ |
| David 2017 | Spain | 22/42 | PE:36.94 ± 12.46 NIP:36.32±10.67 | PE:34  NIP:34 | PE/NIP | PE:172± 8.5 NIP:173±6.7 | PE:65.54±7.08 NIP:65.10±7.24 | 50 minutes/time, 2 times/week, 12 weeks | VAS; RMDQ |
| Young 2012 | Korea | NA | SE:20.1±0.7 OE:20.5±0.5 | SE:15  OE:15 | SE/OE | SE:168.2±9.0 OE:164.5±4.6 | SE:56.9±14.3 OE:54.7±7.6 | 2 times/week, 4 weeks | VAS |
| Cho 2014 | Korea | NA | NA | TC:20  STE:20 | TC/STE | NA | NA | 60 minutes/time, 3 times/week, 4 weeks | VAS |
| Hwangbo 2015 | Korea | 17/13 | CSE:34.5±4.0 MUE:34.0±2.9 | CSE:15  MUE:15 | CSE/MUE | CSE:171.3±5.3 MUE:175.0±4.4 | CSE:62.6±4.6 MUE:75.0±11.6 | 3 times/week, 6 weeks | VAS |
| Young 2015 | Korea | NA | NA | OE:24  CSE:24 | OE/CSE | NA | NA | 50 minutes/time, 3 times/week, 6 weeks | VAS |
| Ui 2015 | Korea | 0/40 | MUE:41.2±5.5 CSE:41.2±6.7 | MUE:20  CSE:20 | MUE/CSE | MUE:161.5±6.0 CSE:159.9±4.7 | MUE:59.7±7.2 CSE:56.6±4.2 | 50 minutes/time, 3 times/week, 6 weeks | ODI |
| Seong 2016 | Korea | 0/40 | OE:69.4±4.1 MAT:70.4±3.2 | OE:20  MAT:20 | OE/MAT | OE:158.1±4.5 MAT:156.3±4.9 | OE:59.2±10.1  MAT: 58.3±5.7 | 3 times/week, 8 weeks | VAS |
| Kwang 2018 | Korea | NA | CSE:43.1±3.7 SE:43.6±4.5 NIP:41.3±3.8 | CSE:10  SE:10  NIP:9 | CSE/SE/NIP | CSE:161.6±3.4 SE:160.0±3.8 NIP:161.1±4.2 | CSE:57.5±6.1 SE:58.7±5.0 NIP:56.2±4.6 | 60 minutes/time, 3 times/week, 12 weeks | NRS; ODI |
| Adamantios 2017 ^77^ | Germany | NA | PT:31.9±6.0 NIP:31.4±5.5 | PT:24  NIP:21 | PT/ NIP | PT:176±9  NIP:174±11.0 | PT:72.1±15.2 NIP:73.4±16.0 | 90 minutes/time, 2 times/week, 13 weeks | VAS |
| Noormohammadpour 2018 | Iran | 0/20 | CSE:43.3±7.5 NIP:41.3±6.4 | CSE:10  NIP:10 | CSE/NIP | CSE:162.5±6.9 NIP:161.5±3.7 | CSE:63.3±4.7 NIP:63.5±5.8 | 8 weeks | VAS; RMDQ |
| Cho 2018 | Korea | 20/16 | MUT:32.7±6.1 CSE:32.4±10.7 | MUT: 20  CSE:19 | MUT/CSE | NA | NA | 3 times/week, 4 weeks | ODI; VAS |
| Yael 2016 | Israel | NA | OE:57.19±8.44 NIP:54.65±6.47 | OE:20  NIP:15 | OE/NIP | NA | NA | 2 times/week, 4 weeks | VAS; RMDQ |
| Ulger 2017 | Turkey | 46/67 | CSE:43.1±14.3 MAT:41.6±12.9 | CSE:56  MAT:57 | CSE/MAT | NA | NA | 60 minutes/time, 3 times/week, 6 weeks | ODI; VAS |
| Martina 2016 | Austria | NA | NIP:29.13±5.93 OE:26.67±6.17 | OE:15  NIP:15 | OE/NIP | NIP:172.67±8.44 OE:177.60±9.98 | NIP:71.67±14.08 OE:69.93±10.59 | 1 times/week, 8 weeks | ODI |
| MSc 2011 | Spain | 13/36 | OE:59.53±5.47 NIP:58.71±4.59 | OE:25  NIP:25 | OE/NIP | OE:158.51±8.84 NIP:156.11±9.44 | OE:78.91±6.53 NIP:72.65±10.61 | 2 times/week, 12 weeks | ODI |
| Kell 2011 | Canada | 78/42 | MUT:42.4±5.6 COR:43.2±5.9 | MUT:60  COR:60 | MUT/ COR: | MUT:177±5  COR:176±5 | MUT:78.4 6±8.8 COR:78.8±6 9.4 | 4 times/week, 12 weeks | ODI; VAS |
| Yoo 2014 | Korea | 47/0 | OE:20.44±1.33 NIP:20.70±1.45 | OE:24  NIP:23 | OE/ NIP | OE;171.52±8.26 NIP:172.40±9.02 | OE:64.69±9.96 NIP:65.80±7.38 | 3 times/week, 8 weeks | VAS |
| Hye 2014 | Korea | 18/0 | OE:20.44±0.27 NIP:20.7±0.37 | OE:9  NIP:9 | OE/NIP | OE:170.56±3.27 NIP:172.40±2.93 | OE:64.41±3.36 NIP:65.80±2.40 | 5 times/week, 8 weeks | VAS |
| Chris 2008 | Netherlands | 44/0 | MUT:44±10 NIP:42±10 | MUT:23  NIP:22 | MUT/NIP | NA | NA | 8 weeks | RMDQ |
| Gur 2003 | Turkey | 15/35 | MUE:35.2±10.51 OE:36.4±9.83 | MUE:25  OE:25 | MUE/OE | NA | NA | 5 times/week, 4 weeks | ODI; VAS |
| Zadro 2019 | Australia | 28/31 | VR:68.8±5.5 NIP:67.8±6.0 | VR:30  NIP:30 | VR/NIP | NA | NA | 3 times/week, 8 weeks | ODI; NRS |
| Kimberly 2009 | USA | 21/69 | Yoga:48.4±1.86 NIP:47.6±1.47 | Yoga:43  NIP:47 | Yoga/NIP | NA | NA | 2 times/week, 24 weeks | ODI; VAS |
| Leonardo 2009 | Australia | 61/93 | MCE:54.6±13.0 NIP:52.8±12.7 | MCE:77  NIP:77 | MCE/NIP | MCE:165±9 NIP:164±10 | MCE:74.5±17.5 NIP:75.9±15.3 | 8 weeks | NRS |
| Kofotolis 2006 | Greece | 0/86 | CSE:40.6±6.4 OE:41.8±7.7 NIP:42.1±8.4 | CSE:28  OE:28  NIP:30 | CSE/OE/NIP | CSE:165.7±8.4 OE:168.6±5.6 NIP:169.2±4.2 | CSE:68.8±3.8 OE:70.1±4.4 NIP:69.6±6.1 | 4 weeks | ODI; NRS |
| Areeudomwong 2017 | Thailand | 11/31 | OE:35.4±10.3 ED:36.2±9.9 | OE:21  ED:21 | OE/ED | OE:162.5±10.5 ED:163.7±9.4 | OE:55.6±7.3 ED:55.8±8.5 | 5 times/week, 4 weeks | NRS; ODI |
| Masharawi 2013 | Israel | 0/45 | CSE:52.45±10.6 NIP:53.6±9.53 | CSE:23  NIP:22 | CSE/NIP | CSE:161.21±6.55 NIP:162.2±4.94 | CSE:70±12.74 NIP:67.75±14.36 | 2 times/week, 4 weeks | VAS; RMDQ |
| Priscila 2015 | Brazil | 14/47 | PT:49.4±12.0 NIP:47.5± 11.9 | PT:31  NIP:30 | PT/NIP | NA | NA | 1 times/week, 12 weeks | RMDQ |
| Kofotolis 2016 | Greece | 0/101 | PE:41.22± 8.49 MUT:39.11±8.68 NIP:42.71±6.10 | PE:36  MUT:37  NIP:28 | PE/MUT/NIP | PE:166±5 MUT:166±7 NIP:162±7 | PE:64.84±8.76 MUT:63.36 ±8.82 NIP:65.00±7.71 | 3 times/week, 8 weeks | NRS; RMDQ |
| Kofotolis 2008 | Greece | 0/88 | CSE:41.0±5.5 MUE:37.5±8.6 PT:41.2±5 NIP:42.2±7.8 | CSE:23  MUE:21  PT:23  NIP:21 | CSE/MUE/PT/NIP | CSE:166.2±0.8 MUE:169.0±0.5 PT:168.5±0.7 NIP:169.9±0.5 | CSE:69.0±3.9 MUE:69.7±6.2 PT:70.0±4.3 NIP:69.1±6.9 | 5 times/week, 4 weeks | NRS; ODI |
| TaeYeong 2020 | Korea | 26/22 | OE:26.0±3.82 CSE:28.79± 9.05 | OE:24  CSE:24 | OE/CSE | NA | NA | 2 times/week, 8 weeks | NRS; ODI |
| Sengul 2021 | Turkey | 23/14 | CSE:40.78±8.8 COR:43.32±8.85 | CSE:21  COR:21 | CSE/COR | NA | NA | 50 minutes/time, 3 times/week, 6 weeks | ODI; VAS |
| Manuela 2007 | Brazil | 75/165 | MUE:54.8±15.3 MCE:51.9±15.3 MAT:54.0±14.4 | MUE:80  MCE:80  MAT:80 | MUE/MCE/ MAT | MUE:164.0±9.9 MCE:163.8±9.2 MAT:163.7±8.4 | MUE:73.3±15.4 MCE:81.0±18.6 MAT:72.8±14.1 | 8 weeks | ODI; VAS |
