# Supplementary material for: Exercise intervention for patients with chronic low back pain: a systematic review and network meta-analysis
Source: Front Public Health. 2023 Nov 17;11:1155225. doi: 10.3389/fpubh.2023.1155225 (PMC10687566; doi:10.3389/fpubh.2023.1155225)
Supplement: Supplementary file 1 [file Data_Sheet_1.zip › Supplementary Appendix 5.PDF]

	Random sequence generation (selection bias)	Allocation concealment (selection bias)	Blinding of participants and personnel (performance bias)	Blinding of outcome assessment (detection bias)	Incomplete outcome data (attrition bias)	Selective reporting (reporting bias)	Other bias
Adamantios 2017	+	+	?	+	+	+	?
Akbari 2008	+	+	?	+	+	+	?
Areeudomwong 2017	+	+	?	?	+	+	?
Bronfort 2011	+	+	−	+	+	+	?
Cai 2017	+	+	−	+	+	+	?
Chatzitheodorou 2007	+	+	?	?	+	+	?
Cho 2014	+	?	?	?	+	+	?
Cho 2014	+	?	?	?	+	+	?
Cho 2015	+	?	?	?	+	+	?
Cho 2018	+	+	?	?	+	+	?
Chris 2008	+	+	−	+	+	+	?
David 2017	+	+	?	+	+	+	?
Demirel 2019	+	+	+	+	+	+	?
Dundar 2009	+	+	?	?	+	+	?
Garcia 2013	+	+	?	+	+	+	?
Goran 2018	+	?	?	?	+	+	?
Gur 2003	?	?	?	?	+	+	?
Hosseinifar 2013	+	+	−	+	+	+	?
Hwangbo 2015	?	?	?	?	+	+	?
Hye 2014	+	?	?	?	+	+	?
Kang 2016	+	?	?	?	+	+	?
Kankaanpää 1999	+	?	?	?	+	+	?
Kell 2009	+	?	?	?	+	+	?
Kell 2011	+	?	?	?	+	+	?
Kim 2014	+	?	?	?	+	+	?
Kim 2015	+	?	?	?	+	+	?
Kimberly 2009	+	?	?	?	+	+	?
Kofotolis 2006	+	?	?	?	+	+	?
Kofotolis 2008	+	+	−	+	+	+	?
Kofotolis 2016	+	+	?	?	+	+	?
Koldaş 2008	+	?	?	?	+	+	?
Kwang 2018	+	?	?	?	+	+	?
Lee 2016	+	?	?	?	+	+	?
Leonardo 2009	+	+	?	?	+	+	?
Liu 2019	+	?	?	?	+	+	?
Macedo 2012	+	+	−	?	+	+	?
Mannion 1999	+	+	+	+	+	+	?
Mannion 2001	+	?	?	?	+	+	?
Martina 2016	+	+	?	?	+	+	?
Masharawi 2013	+	+	−	+	+	+	?
Mohamed 2012	+	+	?	?	+	+	?
Moon 2013	+	+	?	?	+	+	?
MSc 2011	+	+	?	?	+	+	?
Murtezani 2011	+	+	?	?	+	+	?
Murtezani 2015	+	+	−	?	+	+	?
Noormohammadpour 2018	+	+	−	+	+	+	?
Oh 2015	?	?	?	?	+	+	?
Paulo 2007	+	+	?	?	+	+	?
Priscila 2015	+	+	−	+	+	+	?
Roh 2016	?	?	?	?	+	+	?
Ronald 2005	+	?	?	+	+	+	?
Rydeard 2006	+	+	?	?	+	+	?
Sengul 2021	+	?	?	?	+	+	?
Seong 2016	?	?	?	?	+	+	?
Shamsi 2014	+	+	+	+	+	+	?
Shamsi 2016	+	+	?	?	+	+	?
Shnayderman 2013	+	+	?	?	+	+	?
Sipaviciene 2020	+	+	?	?	+	+	?
Suh 2019	+	+	−	+	+	+	?
Szulc 2015	+	+	?	?	+	+	?
TaeYeong 2020	+	+	?	?	+	+	?
Tekur 2008	+	+	−	+	+	+	?
Tekur 2012	+	+	+	−	+	+	?
Torstensen 1998	+	+	−	+	+	+	?
Ui 2015	+	?	?	?	+	+	?
Ulger 2017	+	+	−	+	+	+	?
Unsgaard 2010	+	+	?	?	+	+	?
Valenza 2017	+	+	−	+	+	+	?
Waseem 2019	+	+	−	+	+	+	?
Williams 2005	+	+	?	?	+	+	?
Yael 2016	+	+	−	+	+	+	?
Yoo 2014	+	?	?	?	+	+	?
Young 2012	+	?	?	?	+	+	?
Yozbatiran 2004	+	?	?	?	+	+	?
Zadro 2019	+	+	−	+	+	+	?
