# Supplementary material for: Exercise intervention for patients with chronic low back pain: a systematic review and network meta-analysis
Source: Front Public Health. 2023 Nov 17;11:1155225. doi: 10.3389/fpubh.2023.1155225 (PMC10687566; doi:10.3389/fpubh.2023.1155225)
Supplement: Supplementary file 1 [file Data_Sheet_1.zip › Supplementary Appendix 9.DOCX]

Results of Regression Analysis

|  | Age | Gender |
| --- | --- | --- |
| Pain | -0.45  (-3.44,2.69) | 24.32  (-6.26,105.01) |
| Physical function | -1.51  (-6.08,3.12) | -3.67  (-11.43, 4.20) |
