# Supplementary figures and images for: Exercise intervention for patients with chronic low back pain: a systematic review and network meta-analysis
Source: Front Public Health. 2023 Nov 17;11:1155225. doi: 10.3389/fpubh.2023.1155225 (PMC10687566; doi:10.3389/fpubh.2023.1155225)

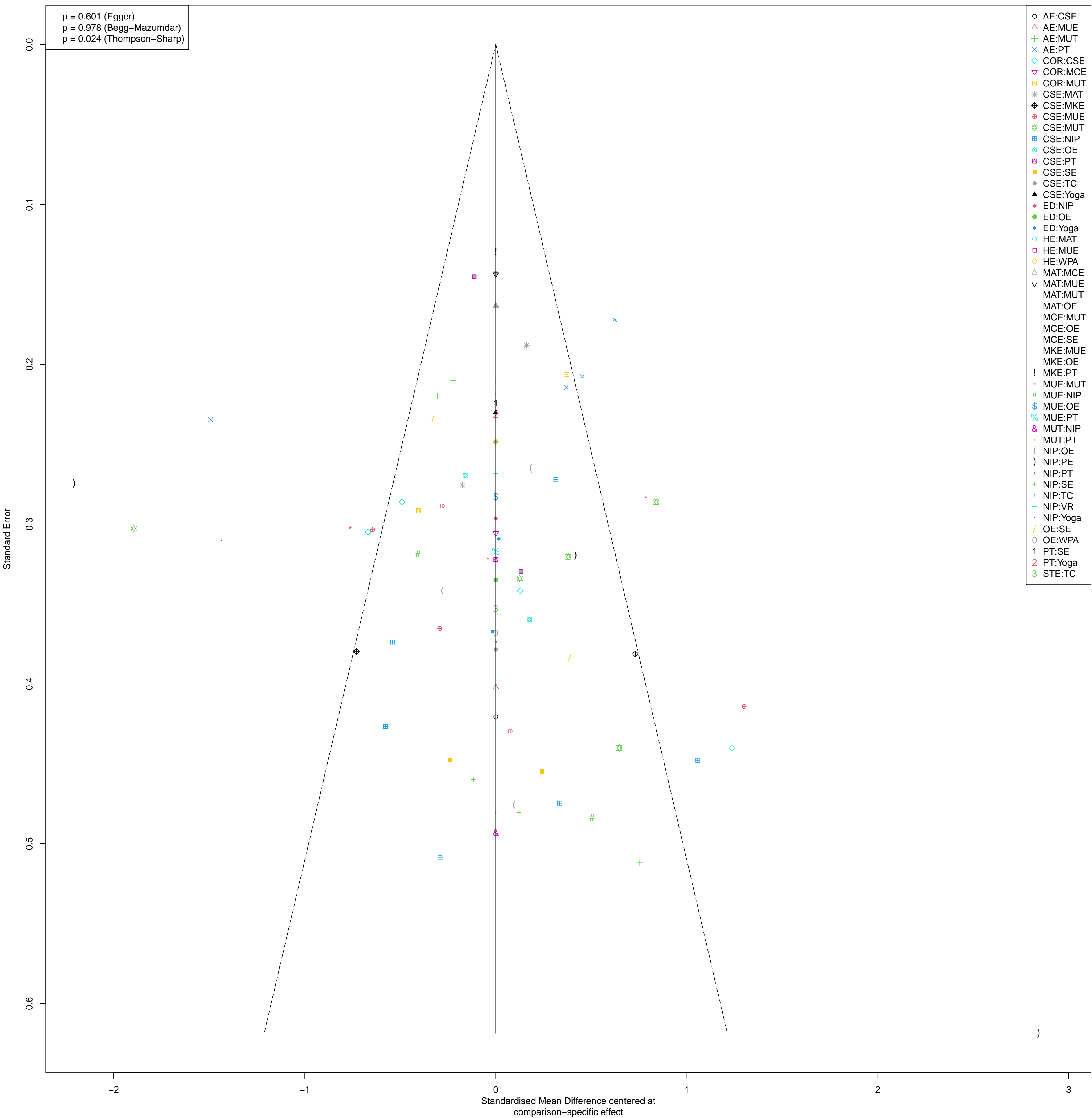

Supplement: Supplementary file 1 [file Data_Sheet_1.zip › Supplementary Appendix 6.1.PDF]

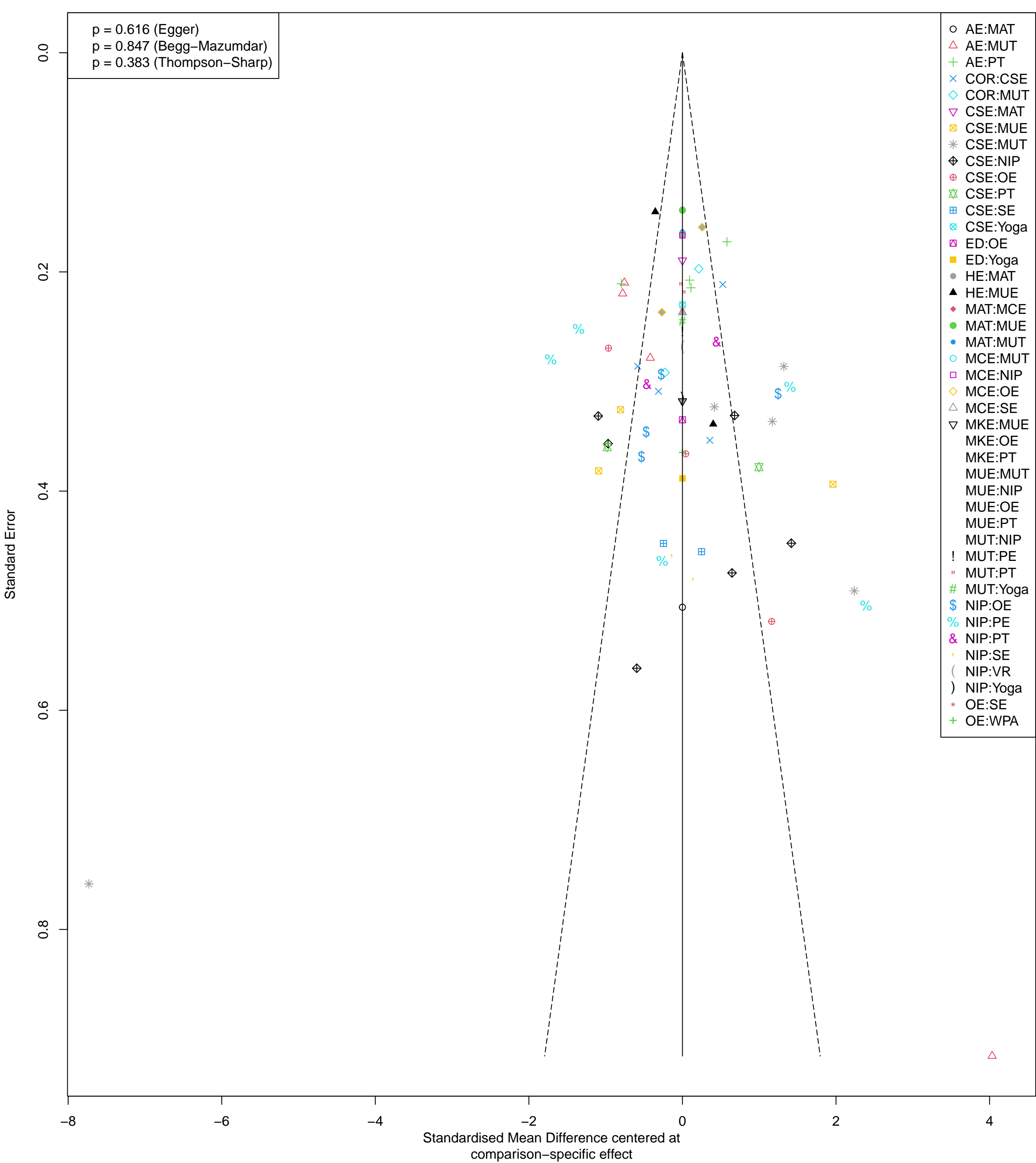

Supplement: Supplementary file 1 [file Data_Sheet_1.zip › Supplementary Appendix 6.2.PDF]

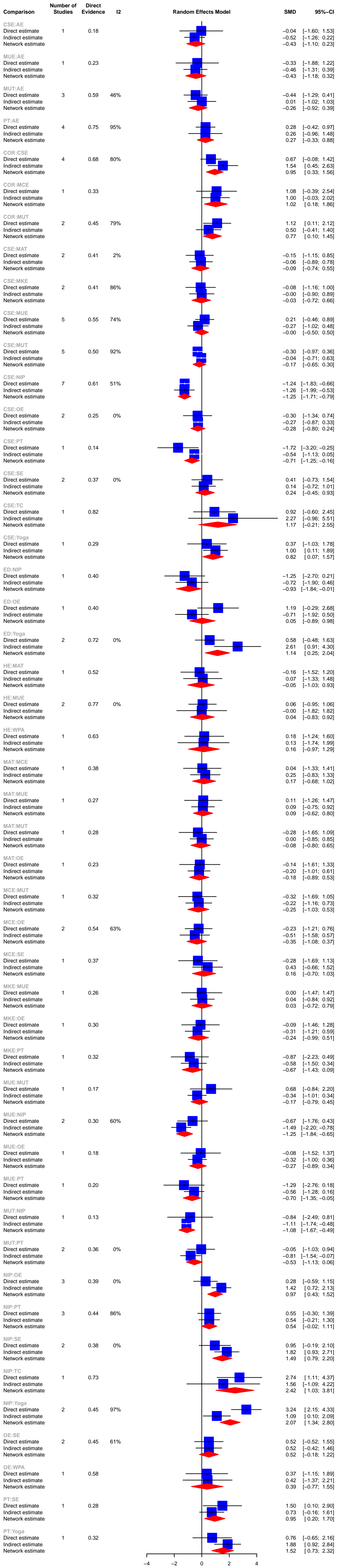

Supplement: Supplementary file 1 [file Data_Sheet_1.zip › Supplementary Appendix 8.1.PDF]

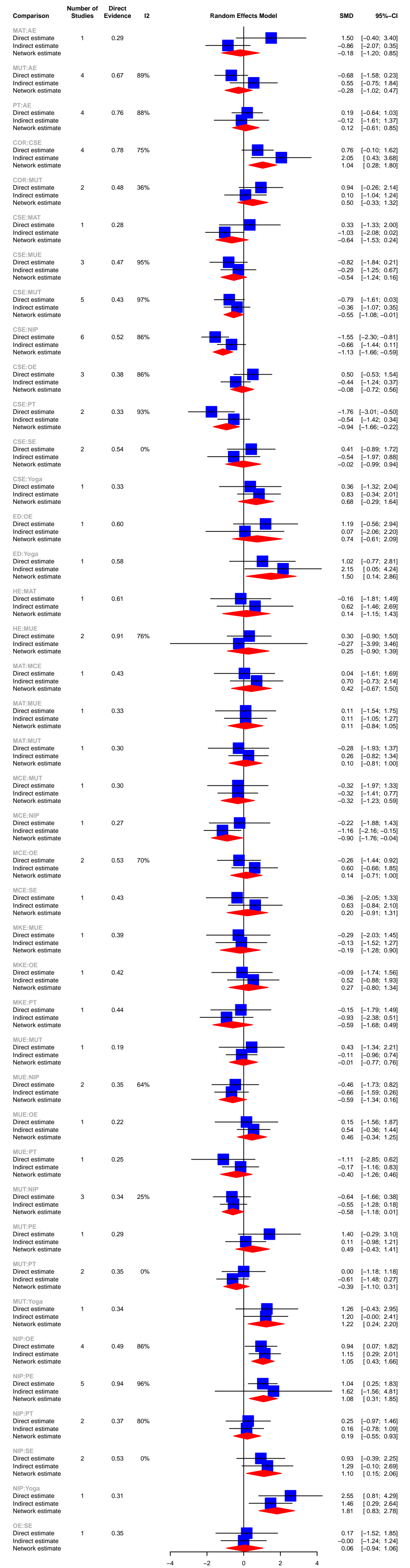

Supplement: Supplementary file 1 [file Data_Sheet_1.zip › Supplementary Appendix 8.2.PDF]
